# Cortical speech envelope tracking reflects lesion-symptom profiles in post-stroke aphasia

**DOI:** 10.1093/braincomms/fcag261

**Published:** 2026-07-07

**Authors:** Guangting Mai, Emily Upton, Timothy D Griffiths, Alexander P Leff, Jennifer T Crinion, Gordon Mills, Douglas Neville, Storm Anderson, Cathy J Price, Ajay D Halai, Martyn I Blairs, Máté Aller, Lucy J MacGregor, Matthew H Davis, Holly Robson

**Affiliations:** Department of Language and Cognition, Psychology and Language Sciences, University College London, London WC1E 6AE, UK; MRC Cognition and Brain Sciences Unit, University of Cambridge, Cambridge CB2 7EF, UK; Department of Language and Cognition, Psychology and Language Sciences, University College London, London WC1E 6AE, UK; Newcastle University Medical School, Newcastle University, Newcastle upon Tyne NE2 4HH, UK; Department of Imaging Neuroscience, UCL Queen Square Institute of Neurology, University College London, London WC1E 6AE, UK; Department of Translational Neuroscience and Stroke, UCL Queen Square Institute of Neurology, University College London, London WC1E 6AE, UK; University College London Hospitals NHS Trust, London NW1 2BU, UK; Institute of Cognitive Neuroscience, University College London, London WC1E 6AE, UK; Department of Language and Cognition, Psychology and Language Sciences, University College London, London WC1E 6AE, UK; Department of Imaging Neuroscience, UCL Queen Square Institute of Neurology, University College London, London WC1E 6AE, UK; Department of Imaging Neuroscience, UCL Queen Square Institute of Neurology, University College London, London WC1E 6AE, UK; Department of Imaging Neuroscience, UCL Queen Square Institute of Neurology, University College London, London WC1E 6AE, UK; MRC Cognition and Brain Sciences Unit, University of Cambridge, Cambridge CB2 7EF, UK; MRC Cognition and Brain Sciences Unit, University of Cambridge, Cambridge CB2 7EF, UK; MRC Cognition and Brain Sciences Unit, University of Cambridge, Cambridge CB2 7EF, UK; MRC Cognition and Brain Sciences Unit, University of Cambridge, Cambridge CB2 7EF, UK; MRC Cognition and Brain Sciences Unit, University of Cambridge, Cambridge CB2 7EF, UK; Department of Language and Cognition, Psychology and Language Sciences, University College London, London WC1E 6AE, UK

**Keywords:** neural tracking, aphasia, speech comprehension, speech envelope, intelligibility

## Abstract

Comprehending connected speech is critical for human interaction and is vulnerable in post-stroke aphasia. Understanding the neural mechanisms underlying impaired speech listening is necessary for accurate and effective assessment and treatment. Neural speech tracking methods offer a window into naturalistic speech processing and may reveal causal contributions to comprehension. EEG was recorded during story listening in 15 people with aphasia with left temporal lesions (temporal group), 14 people with aphasia with left frontal lesions (frontal group) and 15 age- and hearing-matched controls (control group). All participants listened to clear and unintelligible stories (∼12 min per condition). Controls additionally listened to low-intelligibility stories that equated comprehension success to the temporal group. Envelope tracking was measured at syllable- (theta) and multi-syllable- (delta) rates. Neural decoding and encoding analyses measured global (whole-brain) and local (sensor-wise) speech tracking, respectively. Group comparisons used linear mixed-effects regression. Linear and quadratic relationships assessed associations between neural tracking and behavioural measures of comprehension while accounting for covariates. Behavioural measures of comprehension showed the temporal group were significantly impaired compared to both frontal and control groups. Comparison of intelligible and unintelligible speech found that intelligibility affected delta but not theta tracking—suggesting that delta tracking is linked to higher-order linguistic processing and theta tracking reflects sensory responses. Theta and delta envelope decoding reflected group/comprehension status: tracking was reduced in the temporal group compared to the control group, but tracking was not significantly different when control comprehension was behaviour-matched via speech degradation. Delta encoding produced a similarly behaviour-linked pattern, but theta encoding was lesion—rather than behaviour-sensitive, in that reduced tracking was observed in temporo-parietal sensors in both aphasia groups. Theta tracking correlated with comprehension in a lesion-specific manner. Positive correlations between theta tracking and comprehension were found in the temporal group and negative correlations in the frontal and control groups. This produced a quadratic (inverted-U) relationship between theta tracking and comprehension success across all participants. The control-like, negative theta tracking–comprehension correlations in the frontal group are consistent with listening effort effects in which greater task difficulty result in greater tracking. In the temporal group, where comprehension was impaired, better envelope tracking at syllable rates may help build a stable representation of the speech stream supporting phonological and lexical analysis and comprehension. These results indicate that envelope tracking reflects a combination of lesion and symptom profiles and is a viable method for investigating the mechanisms of speech comprehension in aphasia.

## Teaser

Mai, Upton *et al*. report that syllable-rate speech envelope tracking in post-stroke aphasia shows a lesion-specific relationship with speech comprehension. Positive comprehension-tracking correlations following temporal lobe lesions suggest stable envelope representations support the extraction of lexical-semantic information. Negative associations after frontal lesions reflect control-like dynamics linked to listening effort.

## Graphical Abstract

**Graphical Abstract:**
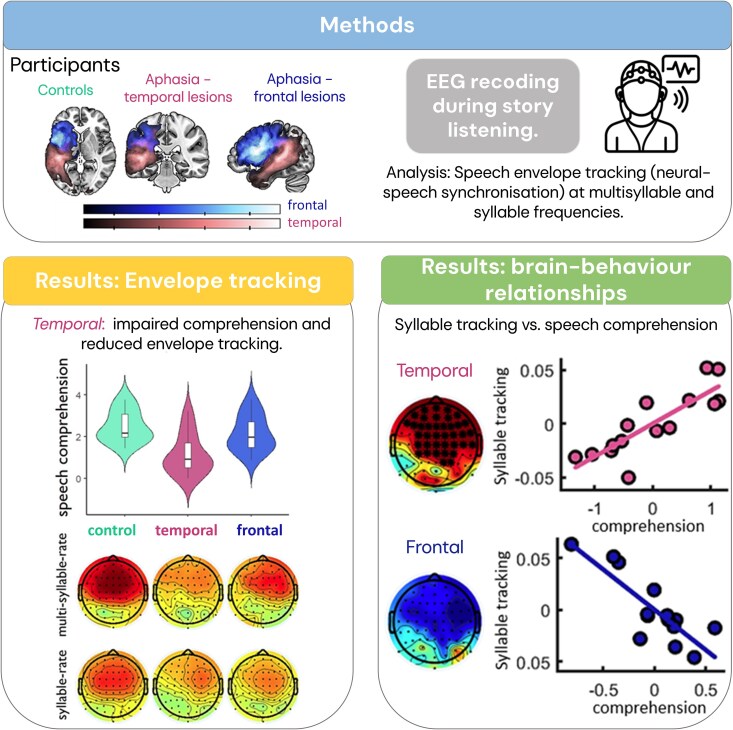

## Introduction

Speech comprehension is frequently disrupted in post-stroke aphasia resulting in feelings of vulnerability and stigmatization.^1^ Impaired comprehension is a negative prognostic indicator^2^ and yet limited therapeutic options are available to clinicians. To compound this challenge, comprehension measured using established behavioural assessments (which assess comprehension at single word or sentence levels) have a limited relationship to the difficulties experienced and reported during continuous speech listening in daily life by people with aphasia (PWA).^1,3^ New methods for evaluating speech processing that can reveal the neural mechanisms responsible for comprehension in PWA are needed if we are to accurately explain comprehension abilities post-stroke and guide rehabilitation.

In this context, observations of neural speech tracking have potentially important application for PWA. Speech tracking methods measure the degree to which cortical activity measured with EEG or MEG is time-locked or phase-locked to ongoing events in a speech stimulus.^4^ Particular attention has been paid to neural responses that track slow amplitude fluctuations in the speech envelope (hereafter envelope tracking). In typical listeners, envelope tracking is a robust effect which is modulated by speech intelligibility^5,6^ and has previously been suggested as a neural marker of speech comprehension success.^7-9^ Moreover, transcranial alternating current stimulation shows that neural responses that track the speech envelope make a causal contribution to speech understanding.^10,11^ Explorations of envelope tracking in PWA may reveal neural mechanisms that contribute to speech comprehension and could provide a target for rehabilitation.

Envelope tracking at different timescales is considered to reflect processing at different linguistic levels. It has been observed that traditional EEG delta and theta frequency bands (typically 0.5–4 and 4–8 Hz, respectively) largely capture a distinction between syllable-level and multi-syllable-level information.^12-15^ As theta rate fluctuations best align to syllabic information, theta envelope tracking may reflect brain activity critical for word-level processing,^16,17^ whereas delta rate envelope signals align to multi-word combinations, syntactic and prosodic information.^13,18,19^ However, due to individual differences in speech production characteristics, generic frequency bands do not always fully reflect stimulus-specific regularities and brain–behaviour relationships may be better revealed using timescales obtained from the speech stimulus.^20^ Theta tracking is predominantly generated in bilateral auditory cortices, consistent with a largely sensory-driven response,^6,21^ whereas delta tracking is similarly generated in auditory regions, but with additional inferior frontal lobe,^18^ and motor generators.^22^ These more widespread neural generators show more pronounced effects of intelligibility,^23^ suggesting that cortical speech tracking at delta frequencies captures a combination of sensory and linguistic processing.^24-26^ Other findings suggest that delta tracking in frontal and motor regions may modulate auditory speech tracking in a top-down manner.^18,27^

Lesion and symptom profiles in post-stroke aphasia indicate a high probability of disrupted speech envelope tracking. Early evidence corroborates this, showing reduced neural tracking of the speech envelope in the broad, delta, theta and gamma bands in PWA compared to healthy controls.^28,29^ In this study, we use EEG to investigate what envelope tracking can reveal about speech comprehension processes during continuous listening in PWA. We aimed to disentangle the cognitive sources and lesion influences on envelope tracking by (i) comparing envelope tracking for intelligible, unintelligible and intermediate-intelligibility speech stimuli (all with preserved speech envelopes), (ii) measuring tracking at stimulus-derived timescales and (iii) extracting global (whole-network) and local (sensor-wise) tracking responses. In addition, by measuring envelope tracking in PWA with either temporal lobe or frontal lobe lesions, we evaluate the specificity of the relationship between tracking, lesion and speech comprehension abilities. Because envelope tracking is a multicomponent and multisource phenomenon, and given that the aphasia envelope tracking literature is in its infancy, we limit ourselves to two broad hypotheses: (i) distinct lesion-symptom profiles will be reflected in envelope tracking and (iii) there will be a statistically significant relationship between envelope tracking and speech comprehension in PWA. Support for these two hypotheses is critical for the future application of envelope tracking to the investigation of speech processing in PWA.

## Methods

### Ethics statement

Ethical approval for this study was granted by the University College London Language and Cognition Ethics Committee (LCD-2021-04). The study was conducted in accordance with the Declaration of Helsinki.

### Participants

Three groups of participants were recruited: (i) *N* = 15 (12 male) participants with aphasia and lesions affecting the left temporal lobe, including the left temporo-parietal junction (temporal group) with limited lesion extension into frontal lobe regions and/or with comprehension impairments in the context of fluent speech; (ii) *N* = 14 (eight male) participants with aphasia and lesions affecting the left frontal lobe (frontal group) with limited extension into the posterior temporal lobe; (iii) *N* = 15 (five male) healthy control participants (control group). Recruitment, exclusion criteria, demographic, screening and diagnostic data are detailed and presented in Supplementary Section 1 and Supplement Tables 1 and 2. Peripheral hearing was screened using pure tone audiometry (air-conduction). ANOVA and *t*-test analyses found no significant group differences in age [*F*(2,41) = 0.37, η2 = 0.018, *P* = 0.70], average bilateral hearing threshold [*F*(2,41) = 0.54, η^2^ = 0.026, *P* = 0.59], time post stroke onset [*t*(27)=−0.41, *P* = 0.68] or lesion volume (see below) [*t*(20.4)=−0.41, *P* = 0.15].

#### Lesion definition

**Figure 1:**
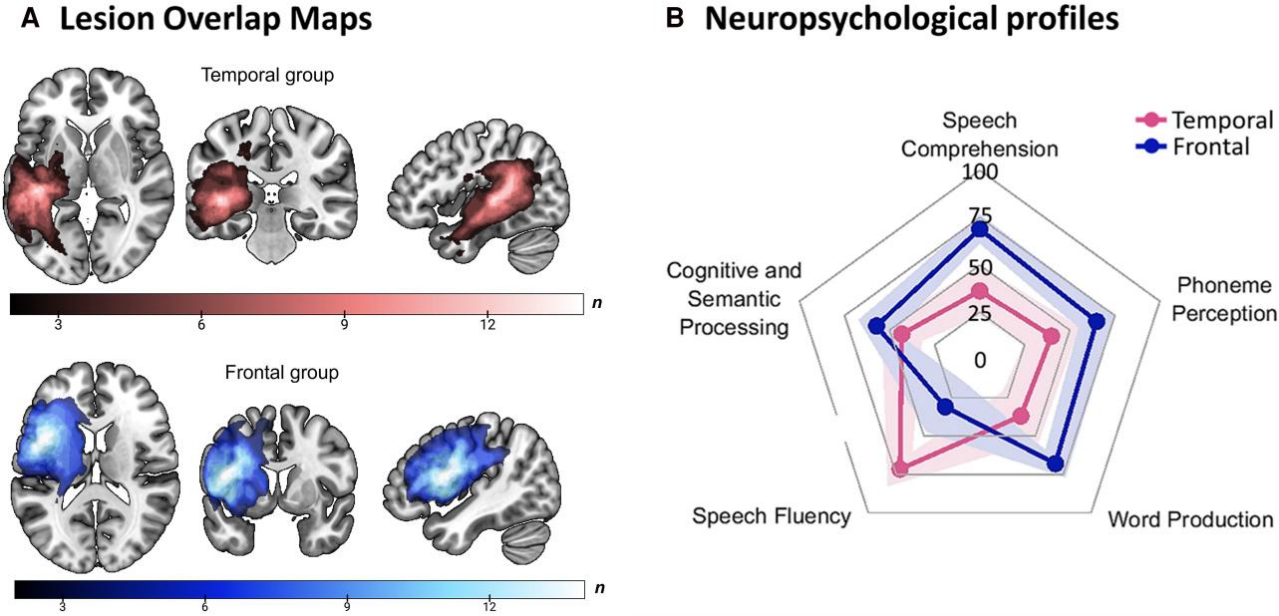
**Aphasia groups’ lesion and neuropsychological profiles.** Sample sizes: *N* = 15 (temporal), *N* = 14 (frontal) and *N* = 15 (controls). **(A) Lesion Overlap Maps.** Lesion overlap maps are displayed separately for the temporal (top) and frontal (bottom) groups, overlaid on the MNI 152 template. Colour bars indicate the number of participants with a lesion at each voxel (*N*). For the temporal group (pink), slices are shown at MNI coordinates *x* = −43, *y* = −29, *z* = 2. For the frontal group (blue), slices are shown at *x* = −45, *y* = 5, *z* = 15. Images produced in MRIcroGL.^32^ (**B**) **Neuropsychological profiles.** Mean percentile rank and 95% confidence intervals are displayed for each group across neuropsychological domains. Percentile ranks were derived from z-scores calculated from the mean and standard deviation of test scores across all participants with aphasia. See Supplementary Table 4 for the tasks contributing to each domain. Image produced by Matlab (R2023b).

Clinical and research brain scan data were obtained for (28/29) participants with aphasia (Supplementary Table 2). Binary lesion images were extracted using automated lesion identification methods based on Seghier *et al.*^30^ and Loughnan *et al*.^31^ Lesion volume was calculated as the number of cubic centimetres identified as lesion. A lesion overlap map is presented in Fig. 1A. Peak lesion overlap in the temporal group involved the planum temporale (*n* = 13; −43,−30,3), no frontal participant displayed lesion to this region. In the frontal group, peak overlap was observed in the pars opercularis (*n* = 14; −46,6,0) and precentral gyrus (*n* = 14; −50,0,15). One temporal participant showed a small lesion extension into the precentral gyrus [although in more superior regions (−34,4,34)]. Greater lesion overlap between the groups occurred in the parietal lobe (peak overlap at −43, −32, 29, *n* = 7 participants from each group), the insular (peak overlap at −40, −9, 1, *n* = 6 participants from each group) and the putamen (peak overlap at −26,4,14, *n* = 4 temporal and *n* = 8 frontal participants).

### Neuropsychological assessment

A neuropsychological battery of language and cognitive assessments was administered over three to four sessions, predominantly online via Zoom, following recommendations in Robson *et al*.^33^ A full description of the assessments and data collection methods is provided in Supplementary Section 2. Language measures assessed: (a) speech comprehension at the single word, sentence and discourse levels; (b) written comprehension at the single word level; (c) word production; (d) speech fluency and (e) phoneme perception. Cognitive measures assessed: (i) non-verbal semantic processing, (ii) phonological short-term memory, (iii) spatial anticipation and (iv) sustained attention; 14/435 assessments were not collected due to a range of factors including participant severity, participant discontinuation, technical failure and participant unavailability. Missing data were imputed, where possible, using the MICE package^34^ (version 3.16.0) in R (version 2023.06.1), see Supplementary Section 3. Data were only imputed if two or fewer datasets were missing for a single assessment. Imputed data are identified in Supplementary Table 4.

## Continuous speech comprehension EEG paradigm

EEGs were recorded while participants listened to semantically and syntactically simple stories from a learn-to-read series (Oxford Reading Tree, Biff, Chip and Kipper stories),^35^ reproduced with permission from the publisher (Supplementary Section 4.1). Participants listened to approximately 12 min of stories per speech condition. Stories were partitioned into blocks of approximately 1.5 min and four comprehension trials were presented after each block. Comprehension trials required participants to judge if a picture was taken from the story (yes–no response). These judgements required comprehension and integration of lexical-semantic story content including characters, contexts and events; 1.5 min of resting-state EEG data were collected before and after the story-listening paradigm. Participants rated their alertness using the sleepy-alert dynamic visual analogue mood scale^36^ immediately before and after EEG data collection.

All participants listened to stories in clear speech and unintelligible speech, created using one-channel noise vocoding. As noise vocoding preserves the speech envelope, this contrast enabled us to disentangle envelope tracking driven by purely sensory responses and those reflecting a combination of sensory and meaningful paralinguistic/linguistic information. Furthermore, as intelligibility and listening effort are known to influence envelope tracking,^6,37-39^ the control group *additionally* listened stories in low-intelligibility speech (four-channel noise-vocoded). By doing this, we aimed to challenge speech perception and comprehension in control participants to match behavioural performance and to equate task difficulty between the control and temporal groups to help in disentangling lesion-related and behaviour-related responses (see Results section). Five experiment versions were developed in which speech condition and story number were counterbalanced and presentation order was pseudo-randomized, see Supplementary Section 4.1. A full description of EEG stimuli, data collection procedure, triggering and timing evaluation are presented in Supplementary Section 4. EEG acquisition and pre-processing are detailed in Supplementary Sections 4 and 5.

### Measuring neural tracking of speech envelopes

#### Determining delta and theta ranges

**Figure 2:**
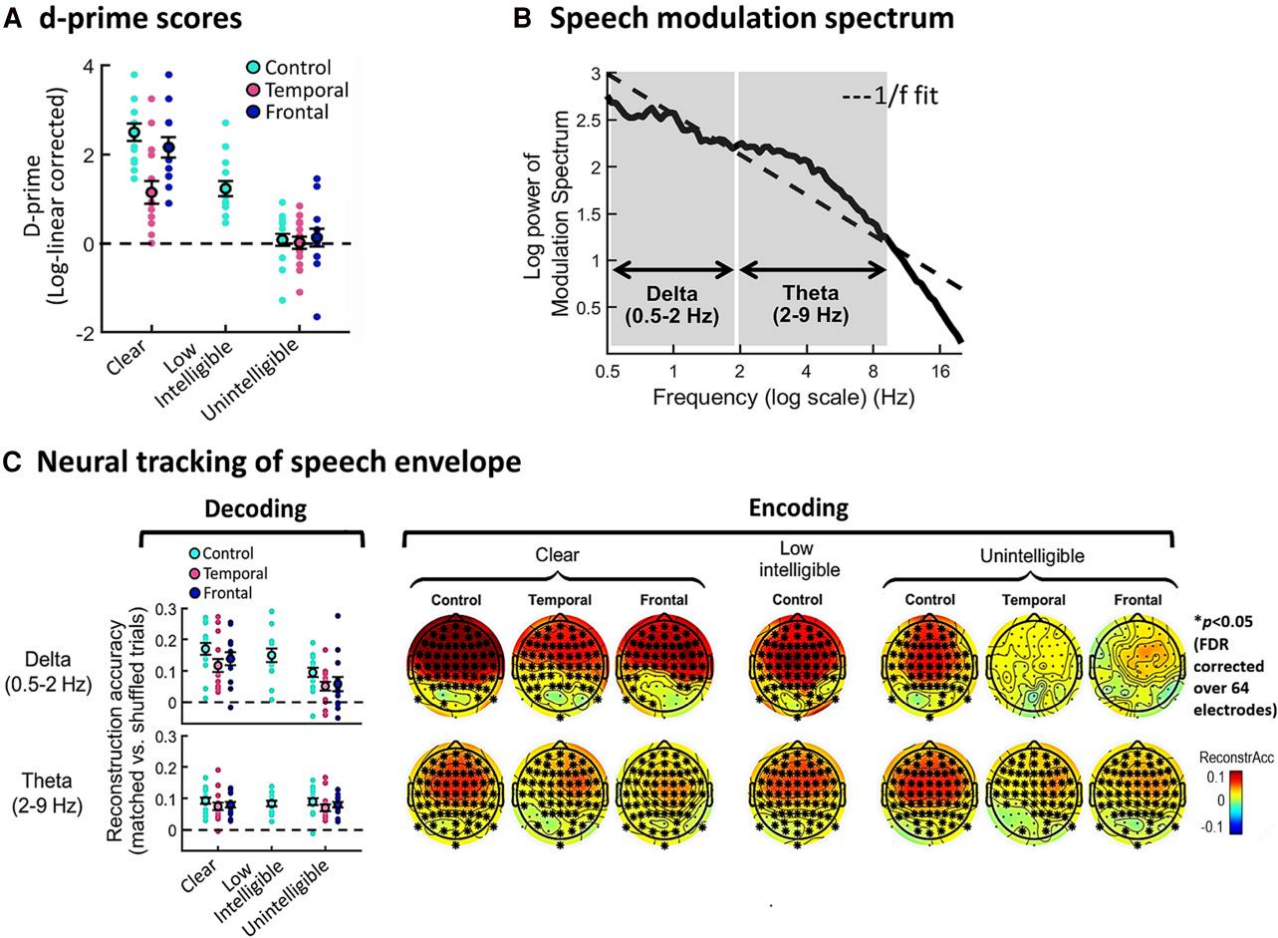
**Behavioural and neural data as functions of group, speech condition and frequency**. Sample sizes: *N* = 15 (temporal), *N* = 14 (frontal) and *N* = 15 (controls). (**A**) EEG story comprehension paradigm d-prime scores. Dots illustrate individual participants’ data with mean values (*d*ʹ, log-linear corrected) of groups shown by black circles (error bars: ±1 SEM). Dashed lines indicate chance-level comprehension. Participants had significant above-chance scores in the clear and low-intelligible conditions (one-sample *t*-tests, *t* > 4.56, *P* < 0.001). (**B**) Modulation spectrum for broadband speech envelopes. The 1/*f* noise profile is fitted over 0.5–20 Hz (log-transformed) illustrating additional modulation power at theta frequencies (2–9 Hz) that correspond to syllable-rate components of speech. (**C**) Normalized decoding (left) and encoding (right) reconstruction accuracy (ReconstrAcc; Pearson’s *r*) (top row: delta 0.5–2 Hz; bottom row: theta 2–9 Hz). Individual participants’ data are shown for decoding with group mean values in black circles (error bars: ±1 SEM). Dashed lines indicate chance-level reconstruction accuracy estimated from 1200 shuffled trials. Decoding accuracy was significantly above chance for all groups, speech conditions and frequencies (one-sample *t*-tests, *t* > 2.58, *P* < 0.023, *P*-values uncorrected). Asterisks in the topographs indicate electrodes where encoding reconstruction accuracy for matched trials was significantly greater than for shuffled trials (i.e. above chance) (one-sample *t*-tests, *t* > 2.18, *P* < 0.05, FDR-corrected over 64 electrodes).

An advantage of cortical speech tracking methods is the ability to simultaneously evaluate the neural representation of speech information at different linguistic timescales under naturalistic conditions. Although generic EEG frequency bands largely capture the distinction between syllable-level and multi-syllable-level information this is not always the case due to individual variation in speech rate.^20^ In this study, we employed a relatively slow speech rate to support all participants to engage with the task (i.e. maintain attention and ensure consistency in cognitive processing throughout) and to avoid floor effects in the temporal group and the control group when listening to low-intelligible speech. Consequently, it was necessary to analyse the story stimuli to derive the frequency bands of speech envelopes for subsequent neural analyses. To determine the frequency ranges, broadband envelopes were obtained by filtering speech at 0.1–5 kHz followed by applying a Hilbert Transform and down-sampled to the same sample rate as EEG (128 Hz). Delta and theta boundaries were determined based on comparing the power spectrum of speech envelopes (i.e. the modulation power spectrum) to a 1/*f* (log scale) noise-profile fitted on the spectrum over 0.5–20 Hz.^40^ We observed elevated modulation power at ∼2–9 Hz above the noise profile (Fig. 2B). Based on this analysis, we thus define delta and theta bands as 0.5–2 and 2–9 Hz, respectively. It has been well established that modulation spectrum reflects syllable rhythms in speech^40^ and the peak of the modulation spectrum (≈4 Hz) best aligned with the average syllable rate (3.36 Hz), consistent with our intended relatively slow speech rate, see Supplementary Table 3. The story materials (taken from a learn to read series) contained a large proportion of monosyllabic words—the average word rate was 2.62 Hz. As such, the theta range (2–9 Hz) will have at least partially captured word-rate envelope fluctuations. Taken together, this confirms that that the traditional delta–theta 4 Hz cut-off would not capture a distinction between syllable-level information and multi-syllable-level information. Both speech envelopes and EEGs were band-pass filtered into the delta and theta ranges by a 2nd-order zero-phase Butterworth filter for analysis, see Supplementary Section 10.

Our delta–theta distinction revealed specific neural outcomes related to the presence of brain lesion and speech comprehension impairments, see Results section. However, due to the unconventional frequency boundary, we replicated our analyses using the broadband envelope (0.5–9 Hz). These results produced a similar overall pattern but statistically weaker evidence, see Supplementary Section 11. This indicates that the stimulus-specific frequency bands afforded greater power to reveal and explain speech comprehension processes in aphasia.

#### Neural speech envelope tracking—decoding and encoding analyses

Neural envelope tracking was measured using complementary encoding and decoding analyses, which quantify the extent to which the EEG signal can be reconstructed from the speech envelope (encoding) and vice versa (decoding). Both methods deliver an outcome measure that is referred to as ‘reconstruction accuracy’; either for neural data (encoding) or for speech envelopes (decoding), respectively. Decoding analyses integrate data from all electrodes, providing an overall measure of neural tracking. In combining data, and down-weighting non-contributing sensors, the influence of lesions on local neural signals can be minimized. In contrast, encoding analyses provide a measure of tracking at the level of individual EEG sensors, thus providing a picture of tracking over space and time. By combining these techniques, we can contrast tracking observed holistically (i.e. how successful is the (residual) network in representing the envelope) with local tracking responses profiles which are expected to deviate due to stroke lesions.

Neural envelope tracking in delta and theta bands was computed using temporal response functions (TRF)^41^ via custom code in conjunction with the mTRF toolbox (https://cnspworkshop.net/resources.html), see Supplementary Section 5.2. In decoding and encoding analyses, EEGs and speech envelopes were segmented into different ‘trials’, each containing approximately one third of a story block (hence, 22–28 s after excluding the first 2 s of each block to minimize onset transient responses). There were thus 24 trials for each speech condition (clear, low-intelligible or unintelligible speech). These trials were partitioned into six subsets and a cross-validation and training-testing procedure was followed using one of the six partitions as testing trials and the remaining trials for training of encoding/decoding analyses. During training, a leave-one-out method was first employed to optimize the ridge parameter *λ*^41^ for each speech condition and individual participant. The full cross-validation procedure is detailed in Supplementary Section 5.3. Decoding/encoding reconstruction accuracy was obtained via this training-testing procedure and averaged over all six partitions. This ensured all trials had equal opportunity to be both training and testing trials, such that outcomes were not biased by outcomes in individual trials. Finally, to measure the reconstruction accuracy against chance-level tracking, we subtracted the observed accuracy from the mean of accuracy estimated based on 1200 random permutations of trial correspondences between stimuli and EEG data during testing.

#### Neural envelope tracking across time

In addition to measuring reconstruction accuracy summed over a time window suitable for detecting speech envelope responses (0–300 ms), we also investigated individual time-lagged accuracy. Specifically, encoding accuracy was calculated at each individual time lag by reconstructing EEG using the TRF (as obtained in the previous training-testing procedures) with a short lag-period centred at that lag. For example, accuracy at 100 ms lag used TRF lags of 92.2, 100 and 107.8 ms, i.e. the closest 3 time points at our 128 Hz sampling rate. Accuracy was further normalized by subtracting shuffled accuracy based on trial permutations for each lag.

### Statistical analyses

#### Behavioural data outcome measures

Comprehension trials during the story listening paradigm were analysed using signal detection methods (d-prime) to evaluate participants’ capacity to discriminate target pictures from non-target distracter items while accounting for yes/no bias. Log-linear correction was applied to account for ceiling performance.^42^ The maximum possible d-prime score, i.e. 100% accuracy, was 3.78. A summary score for comprehension was derived by applying unrotated principal component analysis to all speech comprehension measures from all aphasia participants (temporal and frontal), see Supplementary Section 7 and Supplementary Tables 5 and 6. EEG paradigm d-prime scores and summary neuropsychology comprehension scores were used in evaluating neural-behavioural relationships.

#### LMER of behavioural and neural data

Linear mixed-effects regressions (LMER) were applied to (i) the behavioural data from the EEG paradigm (d-prime scores), (ii) the normalized decoding reconstruction accuracy and (iii) the normalized encoding reconstruction accuracy outcome measures. In each LMER, Group (control, temporal and frontal), Speech Condition (clear/low-intelligible and unintelligible) and Frequency (delta and theta; for reconstruction accuracy only), and the interactions between them, were entered as fixed-effects. Age, hearing (average bilateral pure tone audiometry thresholds at 0.5–4 kHz) and subjective alertness were used as covariates, and participant used as a random-effect variable. Experiment version was additionally used as a random-effect variable for decoding and encoding reconstruction accuracy but not for d-prime due to failure of model convergence. The fixed-effect variables were modelled as factor variables using dummy coding for d-prime and decoding reconstruction accuracy in R (version 2023.06.1 + 524): control group, delta frequency and intelligible speech (clear/low-intelligible) conditions were the reference variables. Electrode-wise LMERs were performed for encoding accuracy in Matlab 2023b. Follow-up time-lagged analyses were conducted *post hoc* for the significant electrodes to further assess at which time points significant effects emerge.

Two models were run for each outcome measure (behavioural d-prime scores; decoding and encoding reconstruction accuracy). The first model used the *stimulus-matched* conditions (clear versus unintelligible speech in all groups) and the second model used the *behaviour-matched* conditions in which the clear speech in the control group was replaced by the low-intelligible speech. False discovery rate (FDR) correction on *P* values for multiple comparisons was applied, see Results section. Alongside testing for statistical significance, Cohen’s *d* (Supplementary Section 8) was further calculated to estimate the effect sizes of significant *post hoc* comparisons.

#### Neural-behavioural relationships

Neural-behavioural relationships were assessed by linearly correlating reconstruction accuracy (delta and theta bands, respectively) in the clear and low-intelligible conditions with the corresponding speech comprehension scores (d-prime) measured during EEG story listening. Pearson’s *r* was obtained for each group after partialling out age, hearing, alertness and lesion volumes (for aphasia groups only). For control participants, we combined the data from clear and low-intelligibility conditions (averaged reconstruction accuracy fitted with the averaged d-prime across conditions) due to the highly similar correlations observed in these two conditions (Supplementary Section 12). For the aphasia participants, we used the neural and behavioural data from the clear speech. Follow-up group comparisons of Pearson’s *r* were performed using Fisher *Z*-tests (Supplementary Section 9). To follow-up on apparently opposite linear correlation direction observed in the sub-group analyses (see Results section), an additional whole-group quadratic fit was conducted to assess the neural-behavioural relationship when considering all groups together. This was performed by linearly fitting reconstruction accuracy with the squared d-prime values (i.e. d-prime^2^ after mean-centring) and partialling out first-order d-prime, age, hearing and alertness. Further follow-up time-lagged analyses were conducted for the significant electrodes by linearly/quadratically fitting the time-lagged encoding accuracy (averaged across electrodes) with d-prime.

The same neural-behavioural analyses were repeated using behavioural data from the neuropsychological measures in the aphasia groups to investigate whether the observed relationships generalized to traditional neuropsychological measures of comprehension. The reconstruction accuracy was linearly and quadratically fitted with a summary comprehension score to reduce the number of comparisons. The summary comprehension score was derived by combining five comprehension measures (see Supplementary Table 4, speech comprehension measures) in an unrotated principal component analysis. Components with eigenvalues > 1 were retained. A single component was identified (eigenvalue = 3.53) accounting for 70.7% of the total variance. All measures loaded strongly and positively on the component (see Supplementary Section 5, Tables 6 and 7).

#### Potential confounds of attention during EEG story listening

Changes in attention were assessed using EEG alpha-band activity^43^ measured as changes in power at 9–12 Hz relative to periods of resting state data collection, see Supplementary Section 6. No statistical impact of Group or Speech Condition on alpha-band activity was found. No statistical changes were observed for LMERs of reconstruction accuracy or for the neural-behavioural relationships by including alpha-band activity as an additional covariate (statistics not reported here). We, therefore, found no evidence that our results were impacted by participants’ level of attention during EEG story listening. This is in line with experimenter observations of attentive listening in all participants.

## Results

### Behavioural results

#### Neuropsychology

Screening and neuropsychological test results and statistical group comparisons are presented in Supplementary Table 4 and summarized in Fig. 1B. The frontal and temporal aphasia groups significantly differed from each other in all domains except for non-verbal semantic processing and non-verbal executive functioning. The temporal group presented with significantly more severe speech comprehension impairments at the single word, sentence and discourse levels. Single-word naming, reading and digit span were also more severely impaired in the temporal than the frontal group; though differences in word repetition did not reach significance. The frontal group had significantly lower speech fluency than the temporal group. Therefore, extending from the inclusion criteria, the aphasia groups presented with distinct neuropsychological profiles, consistent with their lesion profiles and traditional aphasia classifications.

Correlations were performed between the summary neuropsychology comprehension scores and covariates of no interest (age, hearing and lesion volume). In both groups, moderate, significant correlations were observed with age and hearing (*P* < 0.05) and small, nonsignificant effects were observed for lesion volume,^44^  Supplementary Table 7. Variance associated with these factors was partialled out for subsequent analyses.

#### EEG paradigm behavioural scores

Comprehension performance (d-prime for distinguish target and foil pictures) during story listening are presented in Fig. 2A. One-sample *t*-tests confirmed that all groups performed above chance (i.e. d-prime > 0) during clear speech listening, and in low intelligible speech conditions (for control participants) but not in the unintelligible condition (Supplementary Table 8). Partial correlation analysis between d-prime scores in the clear speech condition and a summary neuropsychological comprehension measure in all aphasia participants found a strong relationship (*r* = 0.77, df = 24, *P* < 0.001) when accounting for age, hearing and lesion volume.

Linear mixed-effects regressions (LMER) evaluated the impact of Group and Speech Condition on d-prime scores (Supplementary Table 9). Main effects of Group and Speech Condition as well as an interaction between Group and Speech Condition were observed in both stimulus-matched and behaviour-matched statistical models. *Post hoc* analyses (using the ‘emmeans’ package in R) showed that, in the stimulus-matched model, the control and frontal groups had significantly higher d-prime scores than the temporal group for clear speech with large effect sizes [control versus temporal: *t*(69.1) = 5.0; *P* < 0.001; Cohen’s *d* = 1.56; temporal versus frontal: *t*(69.3) = −3.45; *P* = 0.003; Cohen’s *d* = −1.10] and did not differ from each other [control versus frontal: *t*(68.8) = 1.5; *P* = 0.3]. The behaviour-matched model demonstrated success in using degraded speech to match control performance to the temporal group (listening to clear speech): control d-prime scores did not differ from the temporal group [*t*(69.7) = 0.34; *P* = 0.9] but were significantly lower than the frontal group with a large effect size [*t*(69.7) = −3.1; *P* = 0.008; Cohen’s *d* = −1.24].

### Neural tracking of speech envelopes

#### Robustness of neural decoding and encoding analyses

We first established that neural envelope tracking can be reliably measured overall (decoding analyses) and at individual electrodes (encoding analyses) by comparing matched versus shuffled reconstruction accuracy using paired *t*-tests, Fig. 2C. Above-chance tracking was found for decoding analyses for all groups, speech conditions and frequencies (all *P* < 10^−4^ except: *P* = 0.0019 and *P* = 0.0228 for delta-band unintelligible speech in the temporal and frontal groups, respectively; *P*-values uncorrected). For encoding, above-chance tracking was found in most fronto-temporo-parietal electrodes for all groups and conditions (*P* < 0.05, FDR-corrected over 64 electrodes) except for delta-band accuracy of unintelligible speech in the two aphasia groups.

#### Decoding results

**Figure 3:**
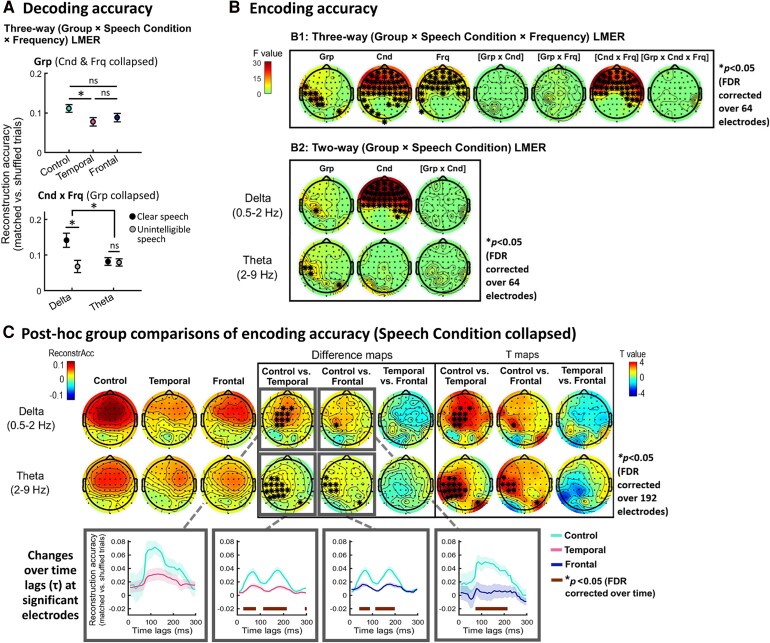
**Statistical analysis of stimulus-matched envelope tracking.** Statistical analysis of decoding and encoding accuracy for speech envelopes in stimulus-matched comparison (clear and unintelligible speech). Sample sizes: *N* = 15 (temporal), *N* = 14 (frontal) and *N* = 15 (controls). **(A) Decoding LMER results.** Three-way [group (Grp) by speech condition (Cnd) by frequency (Frq)] LMER showed: (i) a main effect of group (control > temporal, t = −2.6, *P* = 0.010), upper panel and (ii) a main effect of speech condition (*t* = −4.5, *P* < 0.001) and an interaction between speech condition and frequency (*t* = 3.1, *P* = 0.002) with greater reconstruction accuracy of clear than unintelligible speech only in the delta band (*t* = 7.2, *P* < 0.001), lower panel. The main effect of frequency (delta > theta) is not shown. Asterisks indicate significance (*P* < 0.05). ns = non-significance. Error bars indicate ±1 SEM. **(B) Encoding LMER results.** Three-way LMER (B1) and two-way [group (Grp) and speech condition (Cnd)] LMERs split by Frequency (Frq) (B2) showed similar significant effects of group (specifically focused at left temporal electrodes), speech condition, frequency and interactions between speech condition and frequency. Asterisks indicate significant electrodes (*t* > 4.5 and 7.6 for the three- and two-way LMERs, respectively; *P* < 0.05, FDR-corrected for the corresponding 64 electrodes). **(C) Encoding model *post hoc* comparisons.** Upper panel displays encoding reconstruction accuracy for each group (ReconstrAcc; Pearson’s *r*) and *post hoc* comparisons of group effects on envelope encoding. Asterisks indicate significant electrodes (independent-sample *t*-tests, *t* > 2.9, *P* < 0.05, FDR-corrected for 192 comparisons, i.e. 3 by 64 electrodes). Bottom panel displays time-lagged analyses for electrodes with significant group differences. Shaded areas in the time-lagged figures indicate ±1 SEM. Brown lines indicate time-periods during which reconstruction accuracy significantly differed between groups (independent-sample *t*-tests, *t* > 2.4, *P* < 0.05, FDR-corrected over time).

**Figure 4:**
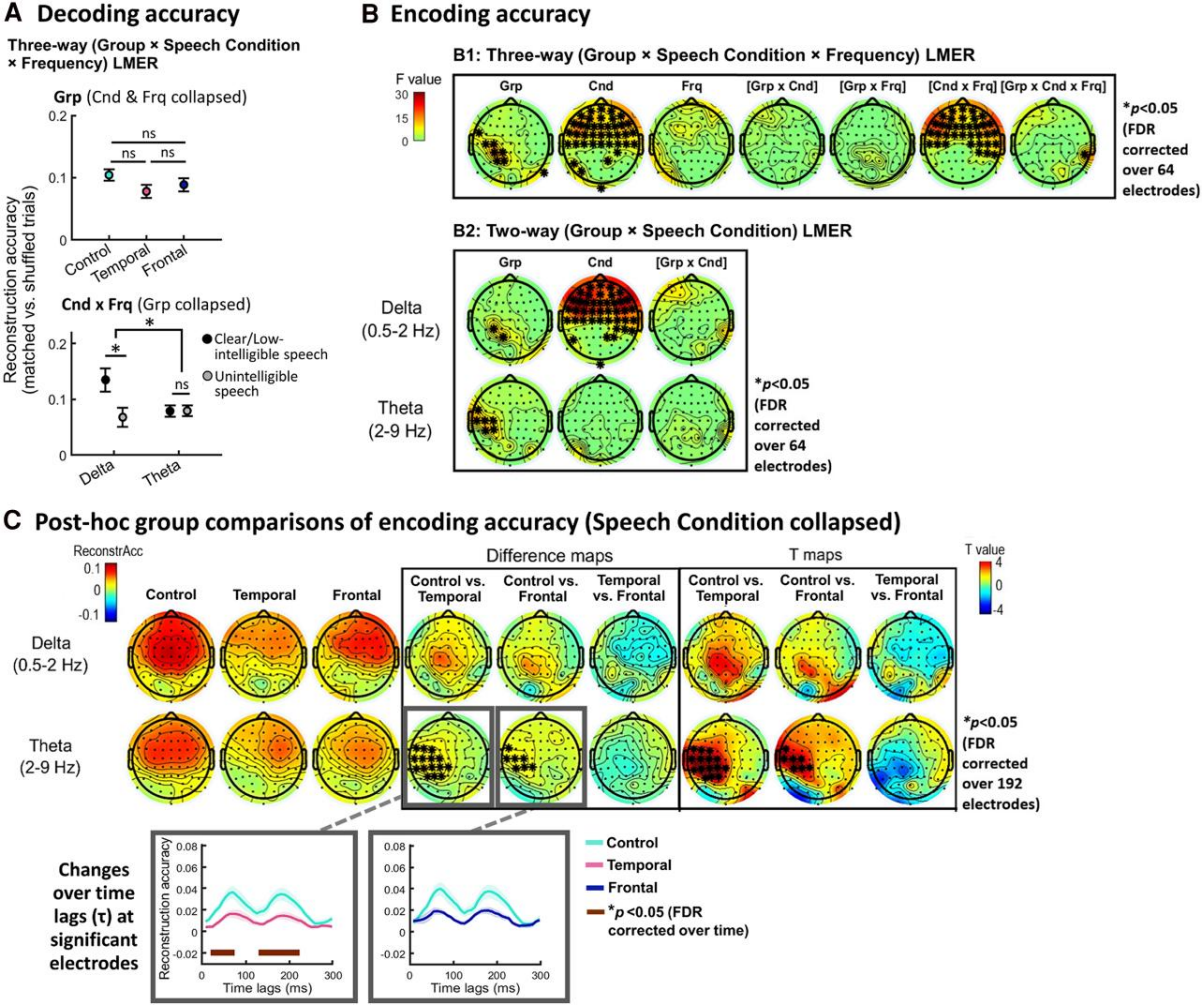
**Statistical analysis of behaviour-matched envelope tracking.** Statistical analyses of decoding and encoding accuracy for behaviour-matched comparison (clear speech in aphasia matched with low-intelligible speech in controls, and unintelligible speech). Sample sizes: *N* = 15 (temporal), *N* = 14 (frontal) and *N* = 15 (controls). **(A) Decoding LMER results.** Three-way [group (Grp) by speech condition (Cnd) by frequency (Frq)]. No group differences are observed (upper)—this contrasts with the stimulus-matched comparison (see Fig. 3A) which found significantly greater tracking in control than temporal participants. The main effect of speech condition (t = −3.6, *P* = 0.001) and interaction between speech condition and frequency (t = 2.5, *P* = 0.012) remained reliable (with greater reconstruction accuracy of clear/low-intelligible than unintelligible speech only in the delta band, t = 6.3, *P* < 0.001). Asterisks indicate significance (*P* < 0.05). ns = non-significance. Error bars indicate ±1 SEM. **(B) Encoding LMER results.** In contrast to decoding results (**A**), significant group effects are observed in left temporal electrodes, similar to those observed in the stimulus-matched comparison (see Fig. 3B). Asterisks indicate significant electrodes (*t* > 4.7 and 4.8 for the three- and two-way LMERs, respectively; *P* < 0.05, FDR-corrected for the corresponding 64 electrodes). **(C) Encoding *post hoc* group comparisons.** Upper panel displays encoding reconstruction accuracy for each group (ReconstrAcc; Pearson’s *r*) and *post hoc* comparisons of group effects on envelope encoding. Group differences did not reach significance in the delta band, in contrast to the stimulus-matched comparison. However, differences remained in the theta band at left temporal electrodes. Asterisks indicate significant electrodes (independent-sample *t*-tests, *t* > 3.0, *P* < 0.05, FDR-corrected for 192 comparisons, i.e. 3 by 64 electrodes). Time-lagged analyses (lower panel) show that significant differences emerged in early time-periods (up to 200 ms lag between the stimulus and response for control versus temporal group comparisons). A similar pattern was observed for the control versus frontal comparison, but this did not reach significance after FDR correction over time. Shaded areas in the time-lagged figures indicate ±1 SEM. Brown lines indicate time-periods during which reconstruction accuracy significantly differed between groups (independent-sample *t*-tests, *t* > 2.4, *P* < 0.05, FDR-corrected over time).

Results of LMERs comparing decoding accuracy are presented in Supplementary Table 10. The stimulus-matched model found a main effect of group, such that decoding accuracy was significantly greater for control participants than the temporal group with a large effect size (β=−0.053, SE = 0.02, *t* = −2.6, *P* = 0.01, Cohen’s *d* = 0.86) but controls did not statistically differ from the frontal group (β=−0.029, SE = 0.02, *t* = −1.2, *P* = 0.18). *Post hoc* comparisons using the emmeans package found no overall difference in decoding accuracy between temporal and frontal groups [*t*(35.4) = −1.02, *P* = 0.57], Fig. 3A. Between-group differences in decoding accuracy were no longer significant in the behaviour-matched model (control versus temporal: β=−0.034, SE = 0.02, *t* = −1.6, *P* = 0.1; control versus frontal: β=−0.01, SE = 0.02, *t* = −0.55, *P* = 0.58), Fig. 4A. Group did not interact with frequency or speech condition and there were no significant effects of covariates (age, hearing and alertness) in either model.

Speech condition significantly interacted with frequency in both stimulus-matched and behaviour-matched models due to intelligibility impacting delta (with large effect sizes) [stimulus-matched: clear > unintelligible, *t*(123) = 7.2, *P* < 0.001, Cohen’s *d* = 0.94; behaviour-matched: clear/low-intelligible > unintelligible, *t*(123) = 6.3, *P* < 0.001, Cohen’s *d* = 0.86] but not theta tracking [stimulus-matched clear versus unintelligible, *t*(123) = 0.27, *P* = 0.99; behaviour-matched: clear/low-intelligible versus unintelligible, *t*(123) = −0.01, *P* = 1]. Therefore, only delta tracking was influenced by the presence of meaningful linguistic information in the stimuli.

#### Encoding results

Encoding analyses were used to determine local neural tracking by observing effects over the scalp topography. Electrode-wise LMERs were performed (three-way LMERs and two-way LMERs split by frequency, see Figs 3B and 4B). *P*-values were FDR-corrected for the corresponding 64 electrodes for LMERs and for 192 electrodes for *post hoc* group comparisons (three between-group comparisons for each of the 64 electrodes) based on independent-sample *t*-tests. Effect sizes were estimated based on outcomes averaged over all significant electrodes.

Encoding results showed both similarities and differences to decoding. As with decoding, stimulus-matched LMERs revealed a significant main effect of group, Fig. 3B. However, encoding differences were seen between control participants and *both* temporal and frontal groups in left fronto-temporal electrodes in both frequency bands (Fig. 3C) (all with large effect sizes, Cohen’s *d* between 1.28 and 1.63), although only a single electrode reached significance for delta encoding in the frontal group. Additionally, group effects *remained* significant in LMERs comparing encoding in behaviour-matched conditions (Fig. 4B) which *post hoc* analyses showed to be due to group differences in theta encoding (large effect sizes, controls versus temporal: Cohen’s *d* = 1.70; controls versus frontal: Cohen’s *d* = 1.53) (delta differences were no longer significant), Fig. 4C. Therefore, locally reduced theta tracking was observed in both aphasia groups irrespective of speech intelligibility or comprehension status. As with decoding, no differences were found between the two aphasia groups in either stimulus- or behaviour-matched analyses.

Follow-up, time-lagged analyses using data from electrodes showing reliable group differences found that encoding differences between control and aphasia groups emerged early (before 100 ms) and extended to ∼200 ms, Figs 3C and 4C, lower panels.

As with decoding, main effects of speech condition on encoding were found in both stimulus- and behaviour-matched models (at bilateral fronto-central-temporal electrodes), and speech condition interacted with frequency due to condition differences (clear/low-intelligible > unintelligible) occurring in the delta (medium to large effect sizes, stimulus-matched: Cohen’s *d* = 0.95; behaviour-matched: Cohen’s *d* = 0.78) but not theta band, Figs 3B and 4B. Again, this showed widespread influence of meaningful speech content on delta tracking, but no measurable influence on theta tracking. There were no significant effects of covariates in either model.

### Neural-behavioural relationships

We tested for linear and quadratic relationships between neural envelope tracking (decoding and encoding reconstruction accuracy) and comprehension measures [d-prime scores for all three groups and summary neuropsychology comprehension scores for the aphasia groups (see Methods section)]. As before, uncorrected *P*-values are reported for decoding analyses and FDR-corrected *P*-values (over 64 electrodes) are reported for encoding results. *P*-values for *post hoc* group comparisons were FDR-corrected to account for the three between-group comparisons for decoding and the 192 comparisons (three between-group comparisons in 64 electrodes) for encoding analyses.

#### Linear correlations

**Figure 5:**
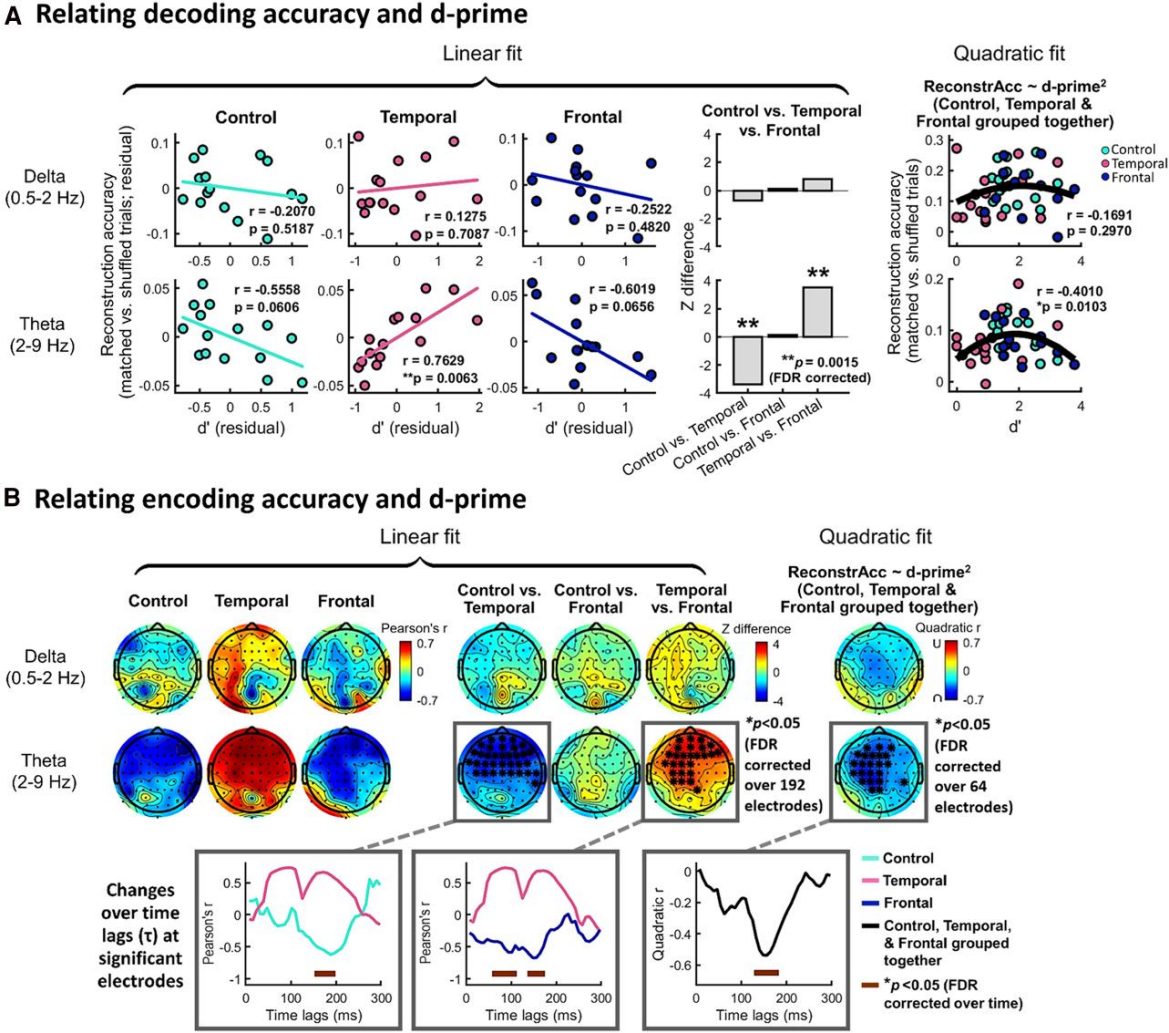
**Relationship between neural envelope tracking and d-prime comprehension measures (intelligible conditions only)**. Sample sizes: *N* = 15 (temporal), *N* = 14 (frontal) and *N* = 15 (controls). (**A**) Decoding accuracy versus d-prime (*d*’). Each dot represents a participant’s data for the residuals of decoding accuracy versus d-prime [d-prime in the control (green), temporal (pink) and frontal (blue) groups, respectively, after partialling out age, hearing, alertness and lesion volume (aphasia groups only)]. Marginally significant partial correlations between decoding accuracy and d-prime were found in the theta band, i.e. significant positive correlations for the temporal group (*r* = 0.763, *P* = 0.006) but marginal negative correlations for the control (*r* = −0.556, *P* = 0.061) and frontal groups (*r* = −0.602, *P* = 0.066). Significant group differences in these correlations (Fisher *z*-tests) with theta decoding were found between control and temporal (*Z* = −3.35, *P* = 0.0015) and between temporal and frontal groups (*Z* = 3.28, *P* = 0.0015). Bar graphs (second right panel) show comparison of correlation coefficients between groups. A significant quadratic relationship (right panel) between reconstruction accuracy (ReconstrAcc) and d-prime was found when including all participants in analysis (*r* = −0.401, *P* = 0.010). Double asterisks indicate *P* < 0.01. (**B**) Encoding accuracy versus d-prime. Upper panel shows partial correlations between electrode-wise encoding accuracy and d-prime scores (left panels), group differences in correlation coefficients (middle panels) and quadratic relationships (right panels). Asterisks indicate significant electrodes with *P* < 0.05, FDR-corrected for 192 comparisons, i.e. 3 by 64 electrodes for group comparisons (control versus temporal: *Z* < −2.41; temporal versus frontal: *Z* > 2.49), and corrected for 64 electrodes for quadratic fits (*r* < −0.37). Lower panels: Time-lagged analyses using data from electrodes showing significant group differences or effects. The brown lines indicate the time lag at which the partial correlation between envelope reconstruction accuracy and d-prime comprehension performance significantly differs between groups (Fisher *z*-tests, control versus temporal: *Z* < −2.68; temporal versus frontal: *Z* > 2.42) and is significant in the quadratic fit (right panel, *r* < −0.40) (all results *P* < 0.05, FDR-corrected over time). See Supplementary Material Fig. 7 for the relationship between neural envelope tracking and d-prime comprehension measures for the clear and low-intelligible conditions in the control group.

**Figure 6:**
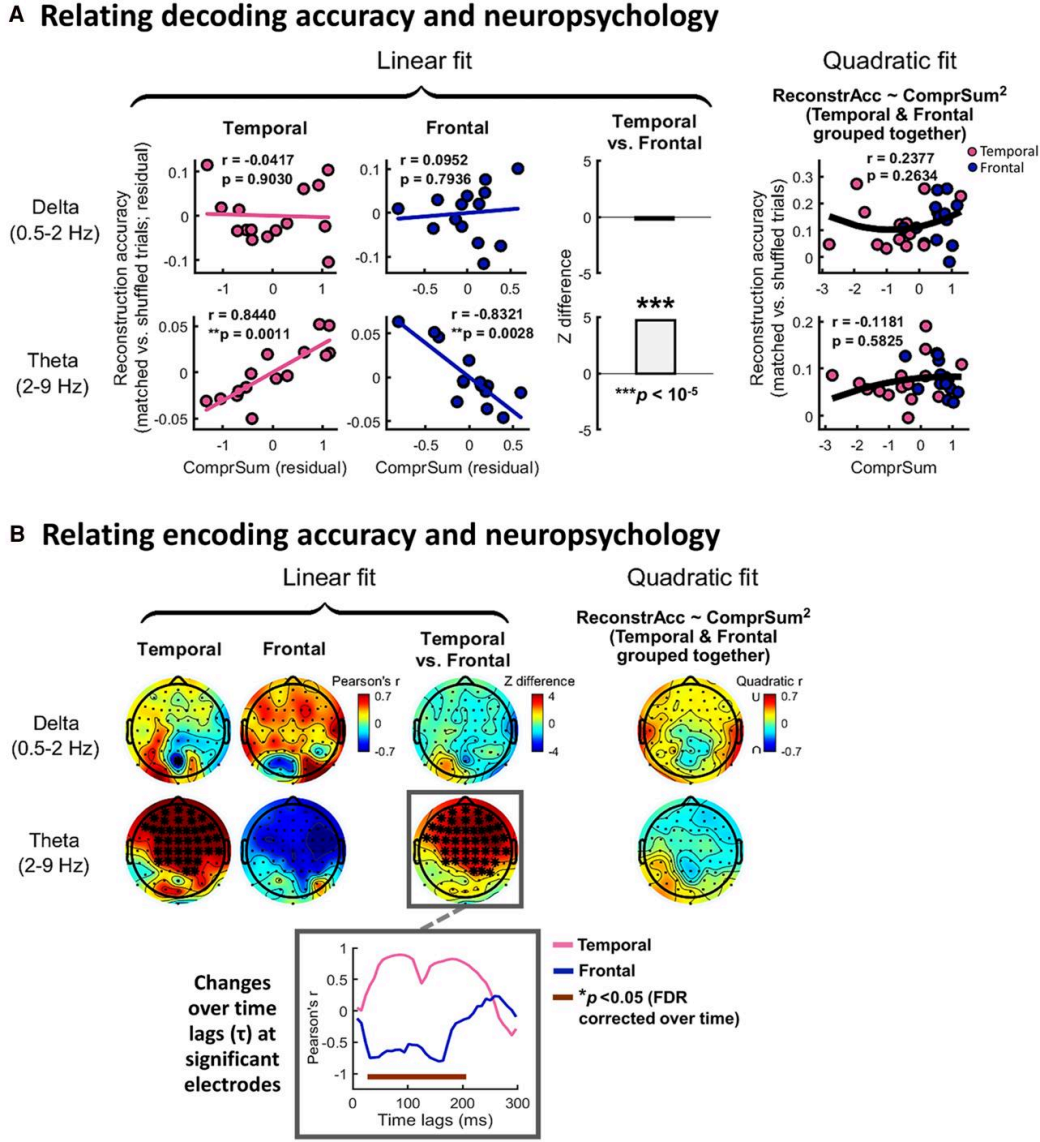
**Relationship between neural envelope tracking and neuropsychology measures of comprehension accuracy for aphasia groups (intelligible speech only)**. Sample sizes: *N* = 15 (temporal) and *N* = 14 (frontal). (**A**) Decoding accuracy (ReconstrAcc) correlations with summary neuropsychological comprehension scores (ComprSum). Each dot represents a participant’s data for the residuals of decoding accuracy versus ComprSum [temporal (pink) and frontal (blue) groups, respectively]. Linear partial correlations and quadratic relationships were fit after partialling out age, hearing, alertness and lesion volume. Strong linear relationships with opposing effect directions for temporal (*r* = 0.884, *P* = 0.001) and frontal groups (*r* = −0.832, *P* < 0.003) were observed as before but not the quadratic relationship was no longer significant. Bar graph displays the significance of comparison of correlation coefficients (Fisher *z*-tests, *Z* = 4.69, *P* < 10^−5^). Double and triple asterisks indicate *P* < 0.01 and *P* < 0.001, respectively. (**B**) Encoding accuracy (ReconstrAcc) versus neuropsychology comprehension summary score (ComprSum). Upper panel displays partial correlations between encoding accuracy and ComprSum group differences in correlation coefficients and quadratic relationships. Asterisks indicate significant electrodes (*P* < 0.05, FDR-corrected for 64 electrodes; significant positive correlations for the temporal group, *r* > 0.652, and significantly greater correlations for the temporal versus frontal group, Fisher *z*-tests, *Z* > 2.15). Lower panel: Time-lagged analyses using data from electrodes showing significant group differences. The brown line indicates the time lag at which partial correlations between envelope reconstruction accuracy and neuropsychological measures of comprehension significantly differed between groups (Fisher *z*-tests, *Z* > 2.18, *P* < 0.05, FDR-corrected over time).

Relationships between comprehension measures and envelope tracking were found in the theta but not delta band, but the direction of these correlations differed between groups, Figs 5 and 6. The temporal group showed significant *positive* correlations between theta decoding reconstruction accuracy and story listening d-prime scores (*r* = 0.7629, *P* = 0.0063), whereas the frontal and control groups showed (marginally significant) *negative* correlations between these variables (control: *r* = −0.5558, *P* = 0.0606; frontal: *r* = −0.6019, *P* = 0.0656). Comparison of correlation coefficients (Fisher *z*-tests, see Supplementary Section 9) confirmed that correlations in the temporal group significantly differed from both the frontal and control groups (who did not differ from each other) (temporal versus control *P* = 0.0015; temporal versus frontal *P* = 0.0015), Fig. 5A. The theta decoding correlations in the temporal and frontal groups were replicated using summary neuropsychology comprehension scores, with the groups showing significant positive and negative correlations, respectively (temporal: *r* = 0.8440, *P* = 0.0011, frontal: *r* = −0.8321, *P* = 0.0028) and for which the correlation coefficients significantly differed (*P* < 10^−5^), Fig. 6A. The relationships between theta tracking and comprehension measures were consistent when evaluated using non-parametric correlations, see Supplementary Section 13 and Supplementary Table 11.

The same pattern of positive and negative correlations was observed in encoding, again for theta tracking only. Strong correlation coefficients were observed in both groups for both d-prime scores and summary neuropsychological comprehension scores. However, after FDR correction, significant electrodes were only identified for summary neuropsychological comprehension scores in the temporal group. As with decoding, significant differences [*P*(corrected) < 0.05] were found between correlation coefficients when comparing the temporal group to the frontal and control groups, Figs 5B and 6B.

#### Quadratic fit

Given the differences in the direction of the correlations, quadratic relationships were also modelled using data from all participants. A significant quadratic relationship was observed between theta reconstruction accuracy and d-prime in decoding (*r* = −0.4010, *P* = 0.0103) and encoding (*P* < 0.05, FDR-corrected over 64 electrodes) at left fronto-centro-temporal electrodes, Fig. 5B. The negative quadratic r indicates an inverted-U curve with initial increases in reconstruction accuracy, largely consisting of temporal group participants, followed by decreases for frontal and control participants with higher d-prime scores. An equivalent quadratic relationship was not found for comprehension scores measured with neuropsychology tasks, potentially because control data were not available for this analysis, which reduces the number and spread of data points observed at the right side of the inverted-U curve (i.e. highly comprehending participants).

#### Time-lagged analyses

Follow-up time-lagged analyses were performed using electrodes which showed significant relationships with d-prime scores (quadratic relationship) and using electrodes for which there was a significant correlation difference between groups (i.e. positive versus negative correlations). Effects emerged at stimulus-response lags of approximately 130–180 ms, Figs 5B and 6B lower panels. This time-course is similar to the characteristics of the group differences in neural envelope tracking shown previously, Figs 3C and 4C.

## Discussion

This study used envelope tracking to investigate neural processes underlying continuous speech comprehension in PWA. In support of our hypotheses, distinct patterns of delta and theta tracking and distinct tracking–comprehension relationships were observed in PWA with temporal and frontal lobe lesions. Most strikingly, the frontal and temporal groups displayed a significant relationship between envelope tracking (at theta rates) and comprehension success, but the direction of this effect differed. This indicates that different mechanisms support speech comprehension across lesion profiles, emphasizing the need to tailor treatments to support underlying processes. Promisingly, these findings suggest that enhancing envelope tracking, for example through non-invasive brain stimulation techniques,^10,45^ may improve comprehension in people with aphasia with temporal lobe lesions.

Envelope tracking simultaneously captures different stages of speech comprehension and it is, therefore, necessary to unpick the different cognitive contributions in order to interpret the processes involved in typical and impaired speech comprehension. Here we created unintelligible speech using one-channel noise vocoding, which disrupts spectral structure while preserving the overall envelope. At the group-level, we found specific intelligibility effects (clear versus unintelligible speech) on delta but not theta tracking; a finding which parallels some (but not all) previous studies.^6,23,24,46,47^ This pattern indicates that our measure of theta tracking primarily captured sensory responses to envelope fluctuations, specifically at syllable timescales, as this measure was unaffected by whether speech was meaningful or not. The strength of delta tracking, on the other hand, was influenced by whether meaningful information was present in the stimulus, although above-chance tracking remained with unintelligible speech in control participants. Together, these findings indicate that, in this study, delta tracking reflected a combination of sensory and above-sensory speech representations, at syntactic and prosodic timescales.^24,26,48,49^ This framework is used to interpret the observed envelope tracking group differences and comprehension correlations.

In line with neuropsychological and lesion profiles, the temporal group had significantly lower story comprehension scores than both the frontal and control groups, which did not differ. This behavioural comprehension pattern was largely reflected in envelope tracking measured using decoding analyses, which index functioning in the residual network.^50^ Delta and theta decoding were significantly reduced in the temporal group in comparison to the control group (decoding success in the frontal group was at an intermediate level). Therefore, the decoding results reflect the observation that the residual auditory and language networks are unable to typically represent speech at sensory or higher-order levels in PWA with impaired comprehension following temporal lobe lesions, but that this ability is better retained in PWA with frontal lobe lesions and better-preserved comprehension. When control participants were behaviourally matched to the temporal group using low-intelligibility vocoded speech, decoding differences were no longer significant. This suggests that these decoding effects cannot easily be accounted for by the presence of stroke lesion (the effect of lesion should still be observed whether behaviour was matched or not) but rather reflect the consistency of neural speech representations.

Encoding analyses complemented decoding analyses by highlighting the topography of tracking and spatial effect of lesions. While delta encoding also showed a comprehension-linked pattern, this was not the case for theta encoding. Both aphasia groups showed reduced theta encoding in left temporal-parietal sensors and this effect emerged early, at <100 ms, and persisted until ≈200 ms. Therefore, reduced theta encoding more clearly reflected the presence of left hemisphere lesions and indicates reduced, inconsistent or absent left hemisphere sensory envelope responses. In the temporal group this can be accounted for as a direct effect of structural damage to the auditory cortices.^24,46,51^ In the frontal group, this may reflect functional depression of auditory regions following lesions to higher-order language areas^52^ and/or reduced predictive processing following damage to linguistic and motor processing in inferior frontal, insular, striatal and motor regions.^20,27,53-56^ Although delta tracking also has a sensory component,^24^ disruption may not be as apparent due to additional contributions to delta activity. The more localized auditory generators of theta tracking^24,46^may combine with lesions in a way that concentrates effects, whereas activity produced by diffuse delta generators may be spread via volume conduction and attenuate the capacity to measure group differences in local auditory responses.

Group differences in theta encoding appear to be lesion but not behaviour-sensitive, in that restricted theta encoding was observed in aphasia irrespective of comprehension status. However, tests of association demonstrated a relationship between theta tracking and comprehension in all three groups. These relationships were observed in both decoding and encoding analyses, although the encoding topography only partially overlapped with sensors demonstrating reduced tracking in aphasia. In other words, explaining behaviour in aphasia requires us to understand how the residual brain network functions rather than merely identifying non-responsive regions.

In all groups, the relationship between theta tracking and comprehension became more reliable after controlling for potential confounds (age, hearing loss, lesion location, subjective alertness and attention). In the aphasia groups, the relationship was observed for both the EEG story comprehension task and a composite measure of clinical and neuropsychological comprehension assessments. Correlations between neural and behavioural measures were observed in both encoding and decoding analyses suggesting that localized differences in neural tracking are not responsible for these associations.

To our knowledge, this is the first demonstration of a relationship between envelope tracking and comprehension in aphasia. Importantly, the direction of the theta-comprehension relationship differed as a function of a combination of lesion location and comprehension impairment: a positive relationship between envelope tracking and comprehension was found in the temporal group and a negative relationship in the control and frontal groups. These findings indicate two different forms of neural engagement with the speech envelope during comprehension. This discovery was not observable through behavioural testing, which only differentiated the groups on severity, thus indicating that speech tracking methods have the potential to reveal symptom and/or lesion-specific mechanisms related to impaired speech processing in aphasia. That the tracking–comprehension correlations were restricted to theta (no delta relationships were observed) aligns with the requirements of the behavioural comprehension assessments used in this study. Our tasks assess the accuracy of lexical-semantic processing, which is dependent on accurately sampling and analysing the speech stream at syllable rates, thus resulting in a strong statistical relationship with theta tracking. In contrast, delta tracking is interpreted to reflect sensory and non-sensory speech representations at prosodic and syntactic timescales. These representations undoubtedly contribute to a holistic interpretation of the story stimuli; however, correlations with behaviour may not be observed because this information is less informative for achieving our comprehension tasks. (Another explanation, however, is that this is because effects after 300 ms were not evaluated).

The positive correlation in the temporal group demonstrates that PWA with greater residual capacity to extract lexical-semantic information show increased time-locking of EEG responses to sensory signals at syllable rates. Plausibly this is a causal relationship, whereby individuals who can more consistently respond to envelope fluctuations build a more stable representation of the speech envelope which, in turn, supports phonological and lexical analysis of the speech stream and provides more accurate input into higher-order language regions. To test this theory, and potentially lay the foundations for novel interventions, manipulation of envelope tracking through electrical stimulation could be explored. It has previously been demonstrated that neurostimulation aligned to the speech envelope can modulate perception of degraded speech in healthy listeners.^10,45^ Even small enhancements of speech comprehension would be functionally beneficial, given the notoriously intractable nature of chronic speech comprehension impairments in PWA with temporal lobe lesions.^57,58^

Crucially, the positive correlation between theta tracking and comprehension in the temporal group could not be accounted for by the presence of a lesion alone and was not replicated in typical listeners through speech degradation. Both the frontal and control groups exhibited negative correlations between theta tracking and comprehension that statistically differed from the positive correlations seen in the temporal group. These findings indicate that the auditory system is behaving typically (i.e. in a control-like manner) in individuals with frontal lobe lesions, despite evidence of diminished theta tracking in left hemisphere temporal-parietal sensors. We interpret these negative correlations between theta tracking and comprehension to reflect increased listening effort for individuals experiencing greater listening difficulty. In this our findings are similar to previous work showing enhanced neural responses for comprehension of spoken sentences that involve increased linguistic complexity, memory load or speech degradation.^59-64^

When data from all three participant groups were combined, a significant quadratic (inverted-U shape) relationship between theta-tracking and comprehension success was observed which we tentatively explain as reflecting non-monotonic neural effects of stimulus engagement (positive correlations) and processing effort (negative correlations).^65,66^ These non-linear properties of theta tracking make it challenging to interpret neural observations of envelope tracking in absolute terms. For individuals in our study with temporal lobe lesions including damage to primary and secondary auditory cortices, better capacity to engage auditory speech responses is associated with greater comprehension success. However, for those individuals with sufficient auditory resources to process speech, the influence of cognitive effort is observed in the negative correlations with comprehension success.

## Conclusions

The results from this study show that envelope tracking reflects comprehension impairments in post-stroke aphasia, but that the magnitude of envelope tracking at an individual level can only be meaningfully interpreted in the context of an individual’s lesion-symptom profile. Future research should aim to disentangle the influence of lesion profile and comprehension impairments on envelope tracking to understand whether the patterns in the current study are driven by a combination of brain and behaviour or whether these are separable factors. Phonetic, phonological, lexical and semantic information all correlate with envelope fluctuations,^67^ making it difficult to disentangle their respective contributions to the present results. Promisingly, recent developments in cortical tracking methods have enabled higher-level acoustic and linguistic representations to be isolated beyond lower-level envelope responses.^68^ The current data demonstrate the potential for cortical tracking to reveal novel insights into speech processing in aphasia and suggest that further research applying these methodological developments could provide a more holistic account of naturalistic speech comprehension.

## Supplementary Material

fcag261_Supplementary_Data

## Data Availability

Preprocessed EEG data (post ICA), restructured EEG, speech envelope and tracking results are available in the UCL data repository at https://doi.org/10.5522/04/31231285. Binary lesion maps and analysis codes are available in an Open Science Framework repository at https://osf.io/hqs2e/. Neuropsychological data are available in Supplementary Materials.
