# Supplementary material for: Cortical speech envelope tracking reflects lesion-symptom profiles in post-stroke aphasia
Source: Brain Commun. 2026 Jul 7;8(4):fcag261. doi: 10.1093/braincomms/fcag261 (PMC13397126; doi:10.1093/braincomms/fcag261)
Supplement: fcag261_Supplementary_Data [file fcag261_supplementary_data.docx]

**Cortical Speech Envelope Tracking Reflects Lesion-Symptom Profiles in Post-Stroke Aphasia**

**Supplementary Materials**

1. Participants
   1. Recruitment, Demographics and Screening

Participants with aphasia were recruited from research registries at University College London^1,2^ and previous research participants known to the authors. Control participants were recruited from contacts of participants with aphasia and contacts of the authors. Diagnostic and screening assessments were conducted over Zoom (<https://zoom.us/>). Participants with aphasia were screened with the Boston Diagnostic Aphasia Examination – Short Form^3^. Control participants were screened over Zoom (<https://zoom.us/>) with the Montreal Cognitive Assessment Audio-Visual Conference version 8.1^4^. Pure-tone audiometry (air-conduction thresholds) was administered in-person to determine bilateral peripheral hearing thresholds for 0.5, 1, 2 and 4 kHz. Hearing screening was unavailable for two participants in the frontal group; these data were imputed using the MICE package^5^ for R (R Core Team, 2024), further details in Supplementary Materials section 3. Inclusion criteria for all aphasia participants were: single symptomatic stroke, over 18 years old. Inclusion criteria for temporal participants were aphasia following stroke affecting the left temporal lobe (including the temporoparietal junction) with limited extension into frontal regions and/or significant comprehension impairments in the presence of fluent speech and without motor symptoms at stroke onset. Inclusion criteria for frontal participants were aphasia following stoke affecting the left frontal lobe with minimum extension into temporal regions and no extension into primary auditory or planum temporale regions. Exclusion criteria for all participants: diagnosis of neurodegenerative disease; significant perceptual impairment that prevented behavioural testing; non-fluent in English (pre-morbidly for stroke participants); non-dominant in English. Exclusion criteria for control participants: clinical range on cognitive screening; significant neurological history. Participants were recruited between September 2023 and October 2024. 15 temporal participants were recruited and completed data collection, none were excluded. 15 frontal participants were recruited and completed data collection, one participant was excluded due to significant extension of stroke into temporal regions. 18 control participants were recruited and completed data collection, three were excluded due to technical error in data collection in which resulted in an inability to re-reference the EEG data.

- 1. Power

Due to a lack of available aphasia tracking data at the time of study design/data collection, power was estimated from an EEG event related potential study exploring speech perception and the relationship with comprehension in a cohort of PWA with similar behavioural profiles to the temporal group^6^. This work found relationships between ERP responses and speech comprehension with effect sizes of r^2^=0.58 and r^2^=0.71, depending on outcome measure. With a significance criterion α = .05 and power = .95, the minimum sample size required to observe a significant correlation was estimated as 11-15 participants using the G*Power 3.1.9.7 software.

|  | **Supplementary Table 1: Participant Demographics** | | | | | |  |
| --- | --- | --- | --- | --- | --- | --- | --- |
| **Group** | **Participant Code** | **Age** | **Handedness** | **Sex** | **Bilingual** | **Average Bilateral Hearing Threshold**  **(db HL)** | **MoCA** |
| Control | 1 | 73 | R | M | no | 31.3 | 26 |
|  | 2 | 75 | R | F | no | 17.5 | 27 |
|  | 3 | 75 | R | F | no | 31.3 | 28 |
|  | 4 | 46 | R | F | yes | 13.8 | 27 |
|  | 5 | 61 | R | F | no | 8.2 | 28 |
|  | 6 | 60 | R | F | no | 36.9 | 27 |
|  | 7 | 58 | R | M | yes | 6.3 | 28 |
|  | 8 | 77 | R | F | no | 30.0 | 27 |
|  | 9 | 62 | R | F | no | 24.4 | 27 |
|  | 10 | 34 | R | M | no | 12.5 | 28 |
|  | 11 | 64 | R | F | no | 6.9 | 27 |
|  | 12 | 63 | R | M | no | 18.1 | 28 |
|  | 13 | 52 | R | F | no | 5.0 | 26 |
|  | 14 | 74 | L | M | yes | 21.3 | 28 |
|  | 15 | 65 | R | F | no | 12.6 | 28 |
|  | Mean(SD) | 62.6(11.9) |  |  |  | 18.4(10.4) | 27.3(0.7) |
| Temporal | 21 | 65 | R | M | yes | 4.4 |  |
|  | 22 | 79 | A | M | no | 35.0 |  |
|  | 23 | 65 | L | F | no | 20.6 |  |
|  | 24 | 45 | R | M | no | 18.1 |  |
|  | 25 | 66 | R | M | no | 62.5 |  |
|  | 26 | 63 | L | M | no | 8.1 |  |
|  | 27 | 63 | R | F | no | 15.6 |  |
|  | 28 | 77 | R | M | no | 35.6 |  |
|  | 29 | 73 | L | M | no | 13.1 |  |
|  | 30 | 63 | R | M | no | 11.9 |  |
|  | 31 | 70 | R | M | no | 43.1 |  |
|  | 32 | 35 | R | M | no | 6.9 |  |
|  | 33 | 84 | R | M | no | 63.8 |  |
|  | 34 | 56 | R | F | no | 14.4 |  |
|  | 35 | 59 | R | M | no | 6.9 |  |
|  | Mean(SD) | 64.2(12.6) |  |  |  | 24.0(19.6) |  |
| Frontal | 41 | 41 | R | M | no | 16.9 |  |
|  | 42 | 77 | R | M | no | 61.9 |  |
|  | 43 | 71 | R | F | no | **22.5** |  |
|  | 44 | 62 | R | F | no | **16.1** |  |
|  | 45 | 75 | L | M | no | 24.4 |  |
|  | 46 | 75 | R | F | no | 21.9 |  |
|  | 47 | 49 | R | M | yes | 13.8 |  |
|  | 48 | 56 | R | F | no | 13.8 |  |
|  | 49 | 47 | R | M | yes | 15.0 |  |
|  | 50 | 61 | R | F | no | 16.3 |  |
|  | 51 | 55 | R | M | yes | 10.6 |  |
|  | 52 | 58 | R | M | no | 20.0 |  |
|  | 53 | 64 | R | M | no | 24.4 |  |
|  | 55 | 55 | R | F | no | 16.25 |  |
|  | Mean(SD) | 60.4(11.1) |  |  |  | 21.0(12.5) |  |
|  | Handedness: A = Ambidexterous; L = Left; R = Right; Average Bilateral Hearing Threshold calculated as the mean air conduction pure tone audiometry thresholds at 0.5, 1, 2 & 4kHz in both ears. *MoCA* = Montreal Cognitive Assessment^7^. Max score = 30, typical cut-off = 26. **Bold text =** value imputed using the MICE R package ^8^ | | | | | | |

| **Supplementary Table 2: Aphasia Participant Screening and Stroke Data** | | | | | | | | |  |
| --- | --- | --- | --- | --- | --- | --- | --- | --- | --- |
|  |  |  |  |  |  | **BDAE Percentile Ranks** | | |  |
| **Group** | **Participant Code** | **Time Post Stroke Onset (months)** | **Brain Imaging** | **Type of stroke** | **Lesion Volume (cm3)** | **Fluency** | **Repetition** | **Comprehension** | **Classification** |
| Temporal | 21 | 204 | 3T MRI | Ischaemic | 108 | 87 | 5 | 10 | WA |
|  | 22 | 106 | CT | Aneurysm | 47 | 100 | 60 | 33 | WA/AA |
|  | 23 | 40 | CT | Ischaemic | 115 | 63 | 5 | 43 | WA/CA |
|  | 24 | 56 | CT | Haemorrhagic | 26 | 100 | 100 | 100 | AA |
|  | 25 | 58 | 3T MRI | Ischaemic | 63 | 48 | 30 | 27 | WA/GA |
|  | 26 | 98 | CT | Ischaemic | 33 | 100 | 5 | 18 | WA |
|  | 27 | 116 | CT | Aneurysm | 108 | 100 | 1 | 5 | WA |
|  | 28 | 14 | CT | Ischaemic | 12 | 100 | 50 | 42 | WA/AA |
|  | 29 | 83 | N/A | Haemorrhagic | **71**^1^ | 100 | <1 | 13 | WA |
|  | 30 | 62 | 3T MRI | Ischaemic | 176 | 63 | 10 | 18 | WA |
|  | 31 | 156 | CT | Ischaemic | 30 | 100 | 1 | 25 | WA |
|  | 32 | 13 | clinical MRI | Haemorrhagic | 65 | 100 | 40 | 33 | WA |
|  | 33 | 103 | 3T MRI | Haemorrhagic | 110 | 100 | 5 | 7 | WA |
|  | 34 | 45 | CT | Ischaemic | 35 | 100 | 60 | 33 | WA |
|  | 35 | 9 | clinical MRI | Ischaemic | 48 | 100 | 75 | 35 | - |
|  | Mean(SD) | 77.5(54.8) |  |  | 69.8(45.0) | 90.7(17.5) | 31.9(32.4) | 29.5(23.0) |  |
| Frontal | 41 | 27 | clinical MRI | Unknown | 49 | 21 | 30 | 57 | BA |
|  | 42 | 54 | CT | Ischaemic | 46 | 67 | 75 | 57 | MNF |
|  | 43 | 29 | CT | Ischaemic | 29 | 40 | 100 | 80 | TMA |
|  | 44 | 129 | 3T MRI | Ischaemic | 155 | 10 | 60 | 100 | BA |
|  | 45 | 60 | CT | Ischaemic | 32 | 63 | 90 | 87 | TMA |
|  | 46 | 158 | 3T MRI | Ischaemic | 182 | 28 | 70 | 71 | BA/TMA |
|  | 47 | 100 | 3T MRI | Ischaemic | 41 | 40 | 100 | 61 | MNF |
|  | 48 | 125 | 3T MRI | Ischaemic | 206 | 37 | 70 | 73 | BA |
|  | 49 | 83 | 3T MRI | Ischaemic | 66 | 67 | 100 | 67 | MNF |
|  | 50 | 40 | CT | Unknown | 85 | 28 | 100 | 50 | MNF |
|  | 51 | 70 | 3T MRI | Ischaemic | 18 | 100 | 45 | 90 | CA |
|  | 52 | 53 | 3T MRI | Ischaemic | 262 | 25 | 100 | 56 | MNF |
|  | 53 | 137 | 3T MRI | Ischaemic + haemorrhagic | 162 | 83 | 100 | 100 | AA/TMA |
|  | 55 | 127 | 3T MRI | Ischaemic | 153 | 100 | 90 | 100 | AA |
|  | Mean(SD) | 85.1(43.8) |  |  | 106.5(78.5) | 50.6(29.4) | 80.7(23.1) | 74.9(17.9) |  |
| Group Difference | t-value(df) | -0.41(27) |  |  | -1.5(20.4) | 4.4(21) | -4.8(27) | -5.9(27) |  |
|  | p-value | 0.68 |  |  | 0.15 | <0.001 | <0.001 | <0.001 |  |
|  | Cohen's d | -0.15 |  |  | -0.57 | 1.7 | -1.8 | -2.2 |  |
| 1 Data imputed for covariate analyses, see Supplementary Materials 3.0  *BDAE* = Boston Diagnostic Aphasia Examination Short Form^3^. N/A = not available; Aphasia Classifications: *WA* = Wernicke's aphasia; *AA* = anomic aphasia; *CA* = conduction aphasia; *GA* = global aphasia; *BA* = Broca's aphasia; *MNF* = mixed non-fluent aphasia; *TMA* = transcortical motor aphasia. | | | | | | | | | |

2.0 Neuropsychological Assessments:

Most neuropsychological assessments were delivered over Zoom (www.zoom.us) following principles described in Robson et al.,^9^. Assessments were administered, controlled and paced by the researcher and presented to participants using the screensharing function. For assessments that required a “pointing response” (e.g. comprehension assessments), participants were given remote control of the researcher’s screen and selected responses by clicking on large response buttons. Two assessments – digit span (phonological short-term memory) and cSART^10^ (sustained attention) – could not be adapted for online administration. Digit span assessments were administered in person. cSART assessments were administered in-person or self-administered using Gorilla software^11^ following face-to-face instructions. Assessment order was pseudorandomised between participants.

1. Language Comprehension:
   1. Single word spoken comprehension was measured with two assessments. (i) The spoken-word picture matching subtest of the Comprehensive Aphasia Test (CAT)^12^; participants selected one of four pictures to match a spoken word. A correct answer scored two points; a correct answer after a delay (7 seconds), self-correction or repetition scored one point. The task was administered using the UCL PLORAS electronic version of the CAT. (ii) A spoken word-picture verification test developed in-house and reported in Robson et al.,^13^. Thirty-five pictures with 100% name agreement were presented followed by a spoken word in three conditions: congruent (e.g. rope-rope), phonologically-related (e.g. rope-robe), semantically-related (e.g. rope-chain). Participants judged if the spoken word matched the picture. A correct answer received one point; a correct answer after two repetitions or more was scored as incorrect. The word-picture verification test was administered in Microsoft PowerPoint.
   2. Single word written comprehension. The word-verification test (above) was presented in a written condition. The written word was visible for one second. Items were presented in a different order from the spoken version. A correct answer received one point; a correct answer after two repetitions or more was scored as incorrect. Administered in Microsoft PowerPoint.
   3. Spoken sentence comprehension was measured with two assessments. (i) A reduced set of thirteen items of Yes-No sentence comprehension questions from the Western Aphasia Battery^14^. Only questions which were suitable for zoom presentation were selected. One point was awarded for each correct answer; two or more repetitions was scored as incorrect. Administered in Microsoft PowerPoint. (ii) Spoken sentence comprehension subtest of the PLORAS electronic version of the CAT; participants selected one of four pictures to match a spoken sentence. A correct answer scored two points; a correct answer after a delay (7 seconds), self-correction or repetition scored one point as per the CAT scoring instructions.
   4. Discourse/continuous speech comprehension. The Discourse Comprehension Test^15^ was adapted following Mackenzie^16^. Five stories were selected and altered to be culturally and lexically appropriate for British English. Each story consisted of approximately 190 words and was followed by eight yes-no comprehension questions presented in a spoken and written modality. A correct answer received one point. Administered in Microsoft PowerPoint.
2. Language Production:

Language production was assessed using subtests from the PLORAS electronic version of the CAT.

- 1. Single word object naming. Participants named 24 items; a correct answer scored two point; a correct answer after a delay (5 seconds) or a self-correction scored one point.
  2. Single word reading. Participants read 24 single words aloud; a correct answer scored two point; a correct answer after a delay (5 seconds) or a self-correction scored one point.
  3. Single word repetition. Participants repeated 16 single words; a correct answer scored two point; a correct answer after a delay (5 seconds), self-correction or item repetition scored one point.
  4. Spoken picture description. Participants were instructed to “describe what is happening in the picture using sentences”. If areas of the picture were omitted, these were pointed to and the assessor prompted the participant by asking “what about this?”. The recorded discourse collected in this subtest was independently assessed by two speech and language therapists to resolve any inconsistencies that arose when assigning speech fluency ratings using the criteria in the Boston Diagnostic Aphasia Examination.

1. Phonological Perception
   1. Nonword Phonological Discrimination from the Psycholinguistic Assessment of Language Processing in Aphasia (PALPA)^17^. Participants judged if two spoken nonwords were the same or different. A correct answer scored one point; two or more repetitions were scored as incorrect. Administered in PowerPoint.
   2. Spoken word discrimination^18^. Participants performed an adaptive 2 alternative forced choice word discrimination task. Trials were presented in XAB format where a reference word accompanied by a picture was presented before two potential target words which were minimal pairs. The participant selected which of the potential targets best resembled the reference word. The acoustic distance between the targets was adaptively modified to change the acoustic difference between the targets. A 3-down, 1-up adaptive procedure was used to converge on an estimate of the proportion of acoustic difference between the targets required for correct identification.
2. Semantic and Executive Processing
   1. Visual Semantic Association was assessed using the Camel and Cactus Test (CCT)^19^. Participants selected which of four semantically related pictures was semantically associated with a probe item. Administered in PowerPoint.
   2. Forward Digit Span^20^. Phonological short-term memory was measured using digit span. Participants repeated numbers presented at one per second starting with a span of two numbers. Span length increased by one number after two correct responses at the previous level. Span was scored as the greatest span length at which both presentations were correct. The digit span task was administered in person and used a pointing response to overcome production impairment.
   3. Spatial Anticipation was assessed using the Brixton spatial anticipation test^21^. Participants are required to identify patterns/rules to anticipate which one of ten numbered circles will be shaded blue on the next page. Each accurate anticipation scores one point. Administered in PowerPoint.
   4. Sustained Attention was assessed using the Sustained Attention to Response Task (cSART)^22^. Participants performed a Go/No-Go task in which they were required to press a button as quickly as possible when they saw a picture of one character (Go trials, n=192) but to withhold their response when presented with a second character (No-Go trials, n=24). Trials were presented in a pseudorandom order.

3.0 Data Imputation

Nine missing data points were imputed using the MICE package (version 3.16.0)^5^ (version 3.16.0; van Buuren and Groothuis-Oudshoorn, 2011) in R (version 2023.06.1): average hearing thresholds (Frontal n=2); discourse comprehension (Frontal, n=1); word discrimination (Temporal n=1; Frontal n=1); written word comprehension (Temporal, n=2) and spatial anticipation (Temporal, n=1; Frontal n=1). Predictive mean matching was used as the imputation method with the following function: mice(data,m=50,maxit=50,meth='pmm',seed=500). Data from 50 iterations was averaged as the final imputed value for each missing data point. All available data with the exception of the cSART sustained attention test were used in the imputation. Missing data from the cSART were not imputed due to a larger proportion of missing datapoints. Imputation was applied to the Frontal and Temporal groups separately.

4.0 EEG Materials and Methods

4.1 EEG Story and Comprehension Stimuli

Ten “level 7” stories from the Oxford Reading Tree, Biff, Chip and Kipper Stories^23^ were selected for use. Level 7 corresponds to approximately a 6-year-old reading age. Stories were semantically and syntactically simple (e.g. contained no passive sentences) and scored an average Flesch Reading Ease score of 95 (SD=4) corresponding to “very easy to read”, Supplementary Table 3. The stories lasted approximately six minutes each and were divided into four approximately equal parts. After each part, participants were presented with four 2 alternative forced choice comprehension trials (16 trials/story). Each comprehension trial consisted of a single picture and participants were asked to verify whether the picture depicted events in the stories using a non-verbal (yes/no) response.  Target/”yes” pictures were illustrations taken from the story book and distracter/“no” pictures were illustrations from another story book in the same reading series of the same illustration style, featuring the same main characters and with at least one overlapping semantic feature (e.g. similar environment or event). For all participants, four stories were presented in clear speech (≈24 minutes in total) and two stories were presented in unintelligible (1-channel noise-vocoded) speech (≈12 minutes in total). The control group additionally listened to four stories (≈24 minutes in total) of low-intelligible speech (4-channel noise vocoding). In the current study, the first two clear speech, the first two low-intelligible stories and both unintelligible stories were analysed so that all speech conditions included similar amounts of data. To ensure any differences in behavioural and tracking success were not due to differences in the difficulty levels of the stories/ comprehension trials, five experimental versions were produced with pseudo-randomised assignment of stories to conditions and pseudo-randomised order of presentation.

| **Supplementary Table 3: EEG Story Stimuli Details and Properties** | | | | | | | | | |
| --- | --- | --- | --- | --- | --- | --- | --- | --- | --- |
| **Story** | **Story Title** | **ISBN-13** | **Publication Year** | **Number of Words** | **Word/ second (Hz)** | **Number of Syllables** | **Syllables/ second (Hz)** | **Flesch Reading Ease** | **Flesch-Kincaid Grade Level** |
| 1 | The Big Breakfast^24^ | 978-0198483298 | *2011* | 852 | 2.42 | 1072 | 3.04 | 98.5 | 1 |
| 2 | The Lightning Key^25^ | 978-0198397038 | 2018 | 814 | 2.52 | 1045 | 3.23 | 94.2 | 2 |
| 3 | Roman Adventure^23^ | 978-0198483151 | 2011 | 869 | 2.52 | 1189 | 3.44 | 89.9 | 2.3 |
| 4 | The Broken Roof^23^ | 978-0198483069 | 2011 | 983 | 2.70 | 1212 | 3.32 | 99.4 | 1.1 |
| 5 | The Time Capsule^26^ | 978-0198300281 | 2015 | 950 | 2.66 | 1285 | 3.60 | 90.2 | 2.7 |
| 6 | A Tall Tale^23^ | 978-0192764300 | 2018 | 891 | 2.54 | 1209 | 3.44 | 89.5 | 2.6 |
| 7 | The Portrait Problem^23^ | 978-0198300243 | 2015 | 965 | 2.63 | 1222 | 3.34 | 95.8 | 1.6 |
| 8 | The Lost Key^23^ | 978-0198483083 | 2011 | 1052 | 2.94 | 1241 | 3.47 | 100 | 0.9 |
| 9 | The Motorway^23^ | 978-0198483205 | 2011 | 885 | 2.58 | 1168 | 3.40 | 97.2 | 1.2 |
| 10 | Lost in the Jungle^23^ | 978-0198483076 | 2011 | 969 | 2.71 | 1201 | 3.36 | 100 | 0.9 |
|  | Mean(SD) |  |  | 923(72) | 2.62(0.14) | 1184 (74) | 3.36(0.15) | 95(4) | 2(1) |
| All stories were authored by Roderick Hunt and Illustrated by Alex Brychta. The Time Capsule and A Tall Tale were co-authored by Paul Shipton. All published by OUP Oxford. Length and readability statistics generated in Microsoft Word. No passive sentences were present in any story. | | | | | | | | | |

4.2 Experimental Procedure

4.2.1 EEG acquisition

Scalp EEGs were recorded by a 64-electrode Biosemi ActiveTwo system (The Netherlands) sampled at 4096 Hz. Common Mode Sense (CMS)/Driven Right Leg (DRL) and bilateral mastoids were ground and reference electrodes, respectively. Direct current offset values were kept below 20 mV. Data were collected in participants’ homes or clinic rooms at University College London.

4.2.2 Training

Following EEG set up and prior to data collection, all participants underwent a short training phase. Training included familiarisation with the main characters and a regularly repeating story event and familiarisation with vocoded speech in the case of control participants. Vocoded speech familiarisation involved control participants listening to typical speech and three levels of vocoded speech (8, 4 and 1-channel) in the presence of a matched written sentence. Participants could listen as many times as they required.  Familiarisation was followed by a short task practice. Aphasia participants practiced with three excerpts (≈15 seconds), in clear speech, from stories not in the experiment. Each excerpt was followed by two practice comprehension trials. Finally, one practice excerpt presented in unintelligible (1-channel vocoded) speech. Controls were presented with the same three practice excerpts, however, the second was immediately repeated in low intelligibility (4-channel vocoded) speech and the last one was repeated in both low intelligibility and unintelligible (1-channel vocoded) speech. Researchers checked that participants understood the task instructions before proceeding to the experiment.

4.2.2 Data Collection

Data collection proceeded as follows:

(1) Pre collection alertness rating

(2) Pre resting state

(3) Story listening and comprehension

(4) Post resting state

(5) Post collection alertness rating

Stimuli were delivered through Microsoft PowerPoint and presented binaurally through a Focusrite Scarlett 2i2 soundcard and ER.3C 50 Ohms insert earphones (Etymotic Research Inc.). Story presentation volume was set by each participant to a comfortable listening level (hence not fixed to a single intensity level) prior to data collection using an excerpt from the experimental material. No adjustments were made to the presentation volume during story listening. The presentation volume was determined by playing pink noise at -18 dBFS RMS through the audio interface and insert headphones. The resulting dB SPL was measured using a B&K 4157 ear simulator, providing dBFS to dBSPL calibration for the playback system. The presentation volume ranged from 58 to 78dB. The median presentation volume for all three groups was 65dB. Envelope reconstruction accuracy has been shown to be insensitive to changes in stimulus intensity even if the changes are large (30dB vs. 60 or 70dB), as long as stimuli used for TRF encoding and decoding analyses are all at a similar intensity level^27^, as was the case in this study.

During resting state data collection and story listening, participants were instructed to look at a blue circular fixation containing an ear icon situated in the middle of the screen which reduced in size until disappearing at the end of the data collection/story block. Story order was determined by experimental version; participants were encouraged to take a break after each story. Three aphasia participants (26, 31, 33), all in the Temporal group, were unable to complete the paradigm in a single session due to fatigue or discomfort. For these participants, a second session was conducted and the data from each session pre-processed separately and combined for analysis. One control participant (15) was presented with the story stimuli in an incorrect order due to incorrect paradigm selection.

4.3 EEG Materials

4.3.1 Stimuli

The stories were professionally recorded by a male British voice actor in a sound-attenuated booth using a RØDE NT1-A 1" condenser microphone (Sydney, Australia), with the audio signal recorded directly to Audacity software running on a Windows 10 PC (Dell Optiplex) via an RME Fireface UC (IMM, Mittweida, Germany) audio interface. Following recording, stimuli were reviewed and adjusted using Praat software (Boersma & Weenink, 2021). Silent periods were reduced to a maximum duration of 500ms and the root mean square amplitude normalised to 70dB.

For the low intelligibility and unintelligible speech conditions, each story was noise-vocoded using Matlab scripts adapted from code used in previous studies from our group^28^. The frequency range of 50–8000 Hz was filtered into 8 or 16 logarithmically-spaced channels based on the work of^29^. Within each channel, the amplitude envelope was extracted by full-wave rectification and application of a lowpass filter with a cut-off of 30 Hz. The resulting channel envelopes were then used to modulate the amplitude of white noise, which was filtered using the same set of logarithmically-spaced filters before the channels were recombined.

4.3.2 Triggering and Timing Evaluation

To correlate the EEG data with features of the stimuli, data acquisition and stimuli presentation should be sufficiently synchronised such that timing errors are of single millisecond order. This is complicated by independent, asynchronous clock sources in the presentation and acquisition system, potentially leading to phase and frequency error. To synchronise EEG and audio data, a timing reference signal was generated by the presentation system and sampled by the acquisition system.

Stimuli consist of a video animation and a monophonic speech recording. A script was developed to embed speech recordings in the left audio channel and synthesise and embed a timing reference signal in the right audio channel of the stimuli videos. The timing reference signal consists of Manchester encoded timestamp data bytes placed at one second intervals. The left audio channel was presented to both ears of the participant whilst the right channel was routed to the digital trigger input of the EEG data acquisition system via a Schmitt trigger circuit. The circuit is effectively a one-bit analogue to digital converter, zero crossings at its analogue input produce digital transitions at its output.

This enables timestamp data to be read and decoded from the EEG data acquisition file. The first timestamp provides a reference point to align the stimuli and EEG data, to an accuracy of one sample period of the acquisition system, thereby solving the problem of phase error. By decoding the final timestamp, the frequency error may also be calculated. The difference of these timestamp values is a measure of the time elapsed according to the stimuli system, and the number of sample periods between the timestamps corresponds to the time elapsed according to the acquisition system. Both the stimuli and acquisition systems employ crystal oscillators for clock generation, which typically have frequency deviations within ±30 parts per million. Over a 90 second period, this could correspond to up to 90 s × 2 oscillators × 30 × 10-6 = 5.4 ms of drift. With the systems deployed in this study, frequency error was measured to contribute approximately 2 ms of drift over the trial period of 90s.

5.0 TRF Methods

5.1 EEG preprocessing

EEG signals were analysed using Matlab R2022b/2023b. The signals were re-referenced to bilateral mastoids, down-sampled to 128-Hz sample rate using a 30th FIR filter (Matlab function ‘decimate’) and segmented. Each segment covered 5 seconds’ pre- and 95 seconds’ post-onset of the corresponding story block. The segmented signals were concatenated and filtered by 2nd-order Butterworth, zero-phase high-pass (0.3 Hz) and notch (48–52 Hz corresponding to the 50-Hz DC-component) filters. Voltages of bad electrodes (excessively large magnitude variations or persistent electrical artefacts over time) were spatially interpolated based on neighbouring electrodes using ‘eeg_interp.m’ in EEGLAB (v9.4.0). EEG artefacts (vertical and horizontal eye movements and motor artefacts) were corrected via Independent Component Analyses (‘Picard’ algorithm in Brainstorm, University of Southern California^30^).

5.2 Decoding and Encoding Analyses

Decoding analysis reconstructs speech envelopes *s(t)* from EEG responses by a linear convolution of decoder *g_n_(t)* and EEG *r_n_(t)* summed over time lags *τ* (0–300 ms) and all electrodes (*n* refers to the *n*th electrode) (Formula (1)). Encoding analysis reconstructs electrode-wise EEG *r_n_(t)* by a linear convolution of TRF (*TRF_n_(t)*) and speech envelopes *s(t)* at variable lags *τ* (0–300 ms) (Formula (2)).

$$s\left( t \right)=\sum_{n} \sum_{\tau} g_{n}\left( \tau\right) r_{n}\left( t+\tau\right)+\varepsilon\left( t \right) \left( 1 \right)$$

$$r_{n}\left( t \right)=\sum_{\tau} {TRF}_{n}\left( \tau\right) s\left( t-\tau\right)+\varepsilon_{n}\left( t \right) \left( 2 \right)$$

*ε(t)* is the error term. The decoder and TRF are estimated using ridge regression:

$\boldsymbol{g}_{\lambda}=\left( \boldsymbol{r}^{T}\boldsymbol{r}+ \lambda\boldsymbol{I} \right)^{-1}\boldsymbol{r}^{T}\boldsymbol{s}\left( 3 \right)$

$\boldsymbol{TRF}_{\boldsymbol{n}\lambda}=\left( \boldsymbol{s}^{T}\boldsymbol{s}+ \lambda\boldsymbol{I} \right)^{-1}\boldsymbol{s}^{T}\boldsymbol{r}_{\boldsymbol{n}} \left( 4 \right)$

In Formula (3), ***r*** and ***s*** are the matrix of lagged time series of EEG and vector of speech envelopes, respectively. In Formula (4), ***s*** and ***r_n_*** is the matrix of lagged time series of speech envelopes and vector of EEG series at the *n*th electrode, respectively^31^. *λ* and ***I*** denote the ridge regression parameter and an identity matrix, respectively. The ridge regularization avoided the ill-posed estimation and overfitting^31^. Both decoding and encoding analyses were conducted at delta and theta bands, respectively.

5.3 Cross-validation and testing

EEGs were segmented into 24 trials in each speech condition (clear, low-intelligible and unintelligible) and were divided into training and testing sets for both decoding and encoding. We partitioned the trials into six subsets (i.e., four trials each subset). A training-testing procedure was repeated for six times, each of which used one of the six subsets as the testing set with the remaining subsets as the training set. Decoding/encoding reconstruction accuracy was obtained using this procedure and the final reconstruction accuracy was averaged over the six repetitions. This ensured that all data had the equal opportunity to be in both training and testing trials to avoid biases from individual trials.

Within each training set, a leave-one-out cross-validation was employed to optimise the ridge parameter *λ* (Crosse et al., 2016). First, one trial within a given training set was selected to be left out as a validator, whilst decoders (for decoding) or TRFs (for encoding) were computed for the remaining trials using *λ* with a range of values (2^-20^, 2^-19^, 2^-18^, …, 2^18^, 2^19^, 2^20^). Second, a different trial was then selected as the validator in the next round of validation and such procedure was repeated until all trials were assigned as validators. Third, cross-validated reconstruction accuracy was obtained via Pearson’s correlations (Fisher-transformed) between reconstructed envelopes/EEGs (obtained using decoders/TRF during each round of validation) and the actual envelopes/EEGs for each validator trial. A *λ* value that yielded the highest mean reconstruction accuracy across all validator trials was then identified for each partition (and also for each electrode during encoding). The final optimal *λ* was selected as follows: for decoding, the optimal *λ* was taken as the one that yielded the highest mean cross-validated reconstruction accuracy across all partitions; for encoding, the optimal *λ* was taken as the mode of the 384 (64 electrodes across six partitions) identified *λ* values, i.e., *λ* with the greatest probability to yield the highest reconstruction accuracy. Such *λ* optimisation was conducted for each participant and speech condition separately.

During the testing, the decoder/TRF was re-computed using the optimal *λ* for each training set and was used to reconstruct speech envelopes/EEGs for the corresponding testing trials. The reconstruction accuracy was estimated as the Pearson’s correlation values (Fisher-transformed) between the reconstructed and actual envelopes/EEGs and averaged over all testing trials and the six partitions.

6.0 Alpha-Band Activity and Attention Effects Evaluation

Attention effects were assessed by EEG alpha-band activity^32^. Specifically, EEGs were Fast Fourier Transformed (FFT) and the logarithmic spectral power was averaged over the alpha range (9–12 Hz) across all story blocks for each speech condition and individual participant. Changes in alpha-band power was then obtained by subtracting the FFT alpha power against that in the two resting-state blocks. We focused on the alpha-band activity at the parieto-occipital electrodes ('P1', 'P3', 'P5', 'P7', 'P9', 'PO7', 'PO3', 'O1', 'Iz', 'Oz', 'POz' 'Pz', 'P2', 'P4', 'P6', 'P8', 'P10', 'PO8', 'PO4' and ‘O2' of the 64-channel ActiveTwo system) which typically reflect auditory attention during speech listening tasks^32^.

Within-subject t-tests showed significant alpha-band activity (negative changes in alpha-band power relative to the resting states) in all groups for all speech conditions (all *p*<10^-5^, uncorrected), consistent with participants attending to the speech stimuli, even in the unintelligible condition. LMERs of alpha-band activity were performed with Group (control, temporal and frontal groups) and Speech Condition (stimulus-matched model: clear vs. unintelligible; behaviour-matched model: clear speech replaced by low-intelligible speech in the control group) as fixed-effect variables, and age, hearing and alertness as fixed-effect covariates. Participant and experiment version were included as random intercepts. Results did not show alpha-band activity was significantly impacted by Group (stimulus-matched: F_(1,81)_=0.9124, *p*=0.3423; behaviour-matched: F_(1,81)_=0.2621, *p*=0.6101) or Speech Condition (stimulus-matched: F_(1,81)_=2.565, *p*=0.1132; behaviour-matched: F_(1,81)_=0.6239, *p*=0.4319). Group did not interact with Speech Condition (stimulus-matched: F_(1,81)_=0.0870, *p*=0.7689; behaviour-matched: F_(1,81)_=1.2108, *p*=0.2744). No significant effects of covariates were found. Furthermore, no statistical changes were found after including the alpha-band activity as an additional covariate for the LMERs of the decoding and encoding reconstruction accuracy or for the neural-behavioural relationships (as described in the main texts).

In sum, we did not find participants’ level of attention statistically varied across groups or speech conditions or changed with individual factors of age, hearing or alertness. Also, there was no evidence showing that the group or speech intelligibility effects on neural envelope tracking or the relationships between neural envelope tracking and behavioural comprehension were statistically impacted by participants’ attention during EEG story listening.

| **Supplementary Table 4: Neuropsychology Assessment Results for Left Temporoparietal and Left Frontal Groups.** | | | | | | | | | | | | | | | | | | |
| --- | --- | --- | --- | --- | --- | --- | --- | --- | --- | --- | --- | --- | --- | --- | --- | --- | --- | --- |
|  |  | Speech Comprehension | | | | | Written Comprehension | Phonological Input Processing | | Language Production | | | | Non-verbal Semantic Association | Cognition | | | |
|  | Measure | sWPV | sWPM | sSV | sSPM | Discourse comprehension | wWPV | Non-word discrimination | Word discrimination | Word repetition | Picture naming | Word reading | Speech Fluency^ |  | Forward Digit Span | Spatial Anticipation | Sustained Attention | |
| Source | | in-house | CAT | WAB | CAT | DCT | in-house | PALPA | in-house | CAT | CAT | CAT | BDAE | CCT | WAIS | Brixton | SART: % correct | SART: n errors |
| Max Score | | 105 | 30 | 13 | 32 | 40 | 105 | 72 |  | 32 | 48 | 48 | 100 | 32 | 9 | 55 | 100 | 0 |
| Typical cut-off | | 94 | 25 | 13 | 27 | 33 | 102 | 61 | 0.34 | 29 | 43 | 45 |  | 26 | 5 |  |  |  |
| Participant IDs for Temporal Group | 21 | *76* | 25 | *10* | *18* | *24** | *76* | 70 | *0.9* | *3* | *3* | *30* | 87 | *24* | *2* | 22 | 99.48 | 1 |
|  | 22 | *91* | *23* | *12* | *17* | 33 | *96* | 63 | *0.62* | *11* | *34* | *24* | 100 | 26 | 5 | 29 | N/A | N/A |
|  | 23 | 94 | 30 | *11* | *18* | *28* | *89* | 69 | *0.56* | *6* | *12* | *17* | 63 | 28 | *4* | 20 | 95.83 | 13 |
|  | 24 | 100 | 27 | *13* | *28* | 39 | 102 | 70 | 0.29 | *32* | *43* | 48 | 100 | 32 | *4* | 43 | 100 | 5 |
|  | 25 | *69* | *17* | *9** | *18* | *27* | *92* | *60* | *0.92* | *20* | *16* | *38* | 48 | *21* | *3* | 31 | 83.85 | 13 |
|  | 26 | *89* | 27 | *9** | *13* | *26** | 104 | *51* | 0.29 | *4* | *30* | *40* | 100 | 29 | *2* | 18 | 98.44 | 6 |
|  | 27 | *92* | *13* | *10* | *9* | *22** | ***85*** | *61* | ***0.7*** | *11* | *2* | *0* | 100 | *15* | *2* | **23.54** | 99.48 | 5 |
|  | 28 | 97 | 28 | *10* | *19* | *31* | *98* | 64 | *0.84* | *28* | *26* | *14* | 100 | 26 | *2* | 19 | 100 | 5 |
|  | 29 | *52** | *7* | *10* | *11* | *24** | *51** | *60* | *0.95* | *0* | *0* | *0* | 100 | *10* | *2* | 27 | N/A | N/A |
|  | 30 | *68* | *20* | 13 | *13* | *34* | *89* | *43* | *0.82* | *16* | *3* | *4* | 63 | *24* | *2* | 28 | 97.92 | 10 |
|  | 31 | *53** | *20* | *8** | *10* | *25** | *94* | *40* | *0.81* | *6* | *15* | *11* | 100 | *24* | *2* | 21 | N/A | N/A |
|  | 32 | 99 | 26 | *11* | *11* | *24** | *96* | 64 | 0.32 | *18* | *39* | *43* | 100 | 32 | 5 | 20 | 99.48 | 0 |
|  | 33 | *54** | *9* | *6** | *7* | *18** | ***89*** | *50* | *0.95* | *2* | *0* | *0* | 100 | *15* | *2* | 23.3 | 70.31 | 12 |
|  | 34 | *90* | *16* | *11* | *17* | *30* | *97* | 64 | *0.54* | 30 | *38* | *24* | 100 | *25* | *2* | 24 | N/A | N/A |
|  | 35 | 102 | 29 | *11* | *24* | *30* | 103 | 69 | 0.27 | 30 | *32* | *42* | 100 | 28 | *3* | 24 | N/A | N/A |
|  | Mean | 81.7 | 21.1 | 10.3 | 15.53 | 27.7 | 90.7 | 59.8 | 0.65 | 14.47 | 19.5 | 22.3 | 90.7 | 23.9 | 2.8 | 24.9 | 94.5 | 7 |
|  | SD | 18.1 | 7.4 | 1.8 | 5.5 | 5.3 | 13.3 | 9.6 | 0.26 | 11.3 | 15.8 | 17.2 | 17.5 | 6.3 | 1.1 | 6.3 | 9.8 | 4.8 |
| Participant IDs for Frontal Group | 41 | 101 | 29 | *10* | *26* | 38 | 104 | 62 | 0.34 | *9* | *37* | *37* | 21 | 29 | *4* | 36 | 97.91 | 16 |
|  | 42 | *67* | *18* | *12* | *20* | 37 | *96* | *58* | *0.85* | *18* | *34* | 46 | 67 | *23* | *5* | 24 | 100 | 4 |
|  | 43 | 95 | 26 | 13 | *24* | *29* | 102 | 62 | ***0.43*** | *23* | *34* | *40* | 40 | 29 | *4* | 26 | N/A | N/A |
|  | 44 | 104 | 30 | *11* | 27 | 34 | 105 | 67 | *0.38* | *10* | *26* | *33* | 10 | 28 | *4* | 32 | N/A | N/A |
|  | 45 | 95 | 27 | 13 | *24* | 33 | *98* | 66 | *0.45* | *24* | *34* | *40* | 63 | *20* | 5 | 26 | 96.88 | 9 |
|  | 46 | *93* | *22* | 13 | *21* | 37 | 103 | 67 | *0.38* | *7* | *26* | *32* | 28 | 27 | *4* | 30 | 96.35 | 22 |
|  | 47 | 95 | *22* | 13 | *23* | *32* | *98* | 70 | *0.69* | *23* | *24* | 46 | 40 | *19* | *4* | 31 | 98.44 | 10 |
|  | 48 | 103 | 28 | *12* | *24* | 34 | 103 | 70 | *0.37* | 30 | 45 | *41* | 37 | 27 | *4* | 28 | 99.48 | 2 |
|  | 49 | 95 | *24* | 13 | *24* | *32* | *98* | 68 | 0.31 | *27* | *38* | 48 | 67 | *25* | 8 | 42 | 99.46 | 3 |
|  | 50 | 100 | 25 | *12* | *21* | **33.3** | *96* | 69 | 0.25 | *24* | *39* | *40* | 28 | 28 | 5 | **27.24** | N/A | N/A |
|  | 51 | 98 | 27 | *12* | *22* | 37 | 100 | 65 | *0.57* | *20* | *32* | *36* | 100 | *25* | *4* | 27 | 100 | 4 |
|  | 52 | 97 | *20* | *11* | *14* | *25** | *94* | *56* | *0.41* | 30 | *29* | *44* | 25 | *23* | *3* | 13 | 97.4 | 24 |
|  | 53 | 99 | 28 | 13 | 29 | 35 | 102 | 68 | *0.38* | *26* | 46 | *44* | 83 | 27 | *4* | 39 | 100 | 1 |
|  | 55 | 103 | 26 | *12* | 28 | 36 | 103 | 71 | *0.52* | 30 | *40* | 46 | 100 | 31 | *4* | 18 | N/A | N/A |
|  | Mean | 96.1 | 25.1 | 12.1 | 23.36 | 33.7 | 100.1 | 65.6 | 0.45 | 21.5 | 34.6 | 40.9 | 50.6 | 25.8 | 4.43 | 28.5 | 98.6 | 9.5 |
|  | SD | 9.1 | 3.5 | 1 | 3.8 | 3.5 | 3.5 | 4.6 | 0.16 | 7.8 | 6.8 | 5.0 | 29.4 | 3.5 | 1.2 | 7.6 | 1.4 | 8.4 |
|  | |  |  |  |  |  |  |  |  |  |  |  |  |  |  |  |  |  |
| Group Difference | t-value  (df) | -2.7  (20.9) | -1.9  (20.4) | -3.4  (27) | -4.3  (27) | -3.6  (27) | -2.7  (16.0) | -2.1  (20.1) | 2.5  (23.5) | -1.9  (27) | -3.4  (19.3) | -4.0  (16.5) | 4.4  (21) | -0.97  (27) | -3.8  (27) | -1.41  (27) | -1.3  (9.4) | -0.82  (18) |
|  | p-value | 0.013 | 0.073 | 0.002 | <0.001 | 0.001 | 0.015 | 0.013 | 0.019 | 0.064 | 0.003 | <0.001 | <0.001 | 0.34 | <0.001 | 0.085 | 0.11 | 0.43 |
|  | Cohen's d | -0.99 | -0.69 | -1.27 | -1.6 | -1.34 | -0.93 | -0.76 | 0.92 | -0.72 | -1.22 | -1.44 | 1.7 | -0.4 | -1.4 | -0.53 | -0.59 | -0.37 |
| Supplementary Table 4 Information: *sWPV* = spoken word-picture verification; *sWPM* = spoken word-picture matching; *sSV* = spoken sentence verification; *sSPM* = spoken sentence picture matching; *wWPV* = written word-picture verification; *CAT* = Comprehensive Aphasia Test^12^; *WAB* = Western Aphasia Battery^14^; *DCT* = Discourse Comprehension Test^15^; *PALPA* = Psycholinguistic Assessment of Language Processing in Aphasia^17^; *CCT* = Camel and Cactus Test^19^; *WAIS* = Welscher Adult Intelligence Scale^33^; Brixton = Hayling and Brixton Tests^21^; SART = Sustained Attention to Response Task^22^; ^ = data replicated from Supplementary Table 2; *italics =* outside normal limits; ***  = chance-level performance; **Bold**  **= imputed data.**  N/A = not available. Data were aggregated for Figure 1B in the following domains: Speech Comprehension: *sWPV, sWPM; sSV; sSPV*; Discourse comprehension. Phoneme Perception: Non-word discrimination; Word discrimination. Word Production: Word repetition, Picture naming; Word reading. Speech Fluency: *BDAE* fluency rating; Cognitive and Semantic Processing: Non-verbal semantic association; Spatial anticipation; Sustained attention (% correct). T-tests were conducted to compare group for each assessment, uncorrected 2-tailed p-values are reported. See Figure 1B in the main text for mean percentile rank and 95% confidence intervals for each group across the neuropsychological domains. | | | | | | | | | | | | | | | | | | |

7.0 Principal Component Analysis: Neuropsychological Comprehension Measures

All neuropsychological measures of comprehension (single word, sentence and discourse) were entered into a principal component analysis (PCA) to derive a summary score for each individual in the Temporal and Frontal groups. The PCA was applied to all stroke participants (i.e. not split by group) to produce a score that was statistically comparable between groups.

The first component produced had an eigenvalue of 3.5 and accounted for 70.7% of the variance, corresponding to overall comprehension severity. The component matrix is presented in Supplementary Table 5 and individual scores in Supplementary Table 6.

| **Supplementary Table 5: PCA Component Loadings for Neuropsychological Comprehension Assessment** | |
| --- | --- |
| **Measure** | **Loading** |
| Spoken word-picture verification | .826 |
| Spoken word-picture matching | .812 |
| Yes/No sentences | .798 |
| Spoken sentence-picture matching | .910 |
| Discourse comprehension | .851 |

| **Supplementary Table 6: Participant Comprehension Severity Scores Derived from PCA** | | |
| --- | --- | --- |
|  | **Participant** | **Comprehension Score** |
| Temporal | 21 | *-0.61* |
|  | 22 | *0.15* |
|  | 23 | 0.15 |
|  | 24 | 1.28 |
|  | 25 | *-1.01* |
|  | 26 | *-0.59* |
|  | 27 | *-1.29* |
|  | 28 | 0.16 |
|  | 29 | *-1.93* |
|  | 30 | *-0.29* |
|  | 31 | *-1.68* |
|  | 32 | -0.40 |
|  | 33 | *-2.78* |
|  | 34 | *-0.39* |
|  | 35 | 0.56 |
|  | Mean(SD) | -0.58(1.04) |
|  |  |  |
| Frontal | 41 | 0.86 |
|  | 42 | *-0.09* |
|  | 43 | 0.56 |
|  | 44 | 0.93 |
|  | 45 | 0.78 |
|  | 46 | *0.62* |
|  | 47 | 0.51 |
|  | 48 | 0.85 |
|  | 49 | 0.62 |
|  | 50 | 0.54 |
|  | 51 | 0.79 |
|  | 52 | -0.48 |
|  | 53 | 1.17 |
|  | 55 | 1.03 |
|  | Mean(SD) | 0.62(0.44) |
| Group Difference | t-value(df) | -4.1(19.1) |
|  | p-value | <0.001 |
|  | Cohen's d | -1.48 |
|  |  |  |

| **Supplementary Table 7: Correlation Matrix between Neuropsychology Comprehension Summary Score and Covariates** | | | | |
| --- | --- | --- | --- | --- |
| Age | Pearson Correlation | -0.408 |  |  |
|  | p-value | 0.028 |  |  |
| Average Hearing Threshold | Pearson Correlation | -0.409 | 0.59 |  |
|  | p-value | 0.028 | 0.001 |  |
| Lesion Volume | Pearson Correlation | 0.035 | 0.006 | -0.134 |
|  | p-value | 0.857 | 0.976 | 0.489 |
|  |  | Comprehension Summary Score | Age | Average Hearing Threshold |

| **Supplementary Table 8: EEG Paradigm d-prime Scores by Group and Condition and One-Sample T-Tests** | | | | |
| --- | --- | --- | --- | --- |
|  |  | **Condition** | | |
|  |  | **Clear** | **Low Intelligible** | **Unintelligible** |
| Control | Mean d-prime (SD) | 2.5(0.75) | 1.2(0.63) | 0.7(0.54) |
|  | t-value | 12.88 | 7.58 | 0.53 |
|  | p-value | <0.001 | <0.001 | 0.6 |
|  | Cohen's d | 3.3 | 2.0 | 0.1 |
| Temporal | Mean d-prime (SD) | 1.1(0.97) |  | 0.01(0.51) |
|  | t-value | 4.57 |  | 0.10 |
|  | p-value | <0.001 |  | 0.92 |
|  | Cohen's d | 1.18 |  | 1.11 |
| Frontal | Mean d-prime (SD) | 2.2(0.86) |  | 0.13(0.74) |
|  | t-value | 9.4 |  | 0.64 |
|  | p-value | <0.001 |  | 0.53 |
|  | Cohen's d | 2.5 |  | 0.17 |

| **Supplementary Table 9: Mixed Effects Modelling of the Impact of Group and Speech Condition on EEG d-prime Scores** | | | | | | | | | | | | | | |
| --- | --- | --- | --- | --- | --- | --- | --- | --- | --- | --- | --- | --- | --- | --- |
|  |  | **Model 1: Speech Matched: Clear vs. Unintelligible** | | | | | |  | **Model 2: Behaviour Matched: Clear/Low Intelligible vs. Unintelligible** | | | | | |
|  |  | **Fixed Effects** | | | | | |  | **Fixed Effects** | | | | | |
|  |  | **Est/Beta** | **SE** | **95% CI** | | **t** | **p** |  | **Est/Beta** | **SE** | **95% CI** | | **t** | **p** |
| Intercept |  | 3.70 | 0.70 | 2.39 | 5.01 | 5.25 | <0.001 |  | 2.38 | 0.68 | 1.11 | 3.65 | 3.48 | <0.001 |
| Control vs. Temporal |  | -1.38 | 0.27 | -1.89 | -0.87 | -5.03 | <0.001 |  | -0.11 | 0.27 | -0.61 | 0.38 | -0.43 | 0.67 |
| Control vs. Frontal |  | -0.42 | 0.28 | -0.94 | 0.10 | -1.49 | 0.14 |  | 0.85 | 0.27 | 0.34 | 1.36 | 3.10 | 0.00 |
| Speech Condition (Intelligible vs. Unintelligible) |  | -2.42 | 0.22 | -2.85 | -1.99 | -10.87 | <0.001 |  | -1.16 | 0.22 | -1.58 | -0.73 | -5.25 | <0.001 |
| Group (Control vs. Temporal) x Speech Condition |  | 1.29 | 0.31 | 0.68 | 1.90 | 4.09 | <0.001 |  | 0.02 | 0.31 | -0.58 | 0.63 | 0.08 | 0.94 |
| Group (Control vs. Frontal) x Speech Condition |  | 0.39 | 0.32 | -0.23 | 1.01 | 1.22 | 0.23 |  | -0.87 | 0.32 | -1.49 | -0.26 | -2.75 | 0.01 |
| Age |  | -0.02 | 0.01 | -0.03 | 0.00 | -1.68 | 0.10 |  | -0.01 | 0.01 | -0.03 | 0.00 | -1.48 | 0.15 |
| Hearing |  | 0.01 | 0.01 | -0.01 | 0.02 | 1.19 | 0.24 |  | 0.01 | 0.01 | -0.01 | 0.02 | 1.15 | 0.26 |
| Subjective Alertness |  | -0.01 | 0.01 | -0.02 | 0.01 | -0.83 | 0.41 |  | -0.01 | 0.01 | -0.02 | 0.01 | -1.02 | 0.31 |
|  | | | | | | | |  |  |  |  |  |  |  |
|  |  | Random Effects | | | | | |  | Random Effects | | | | | |
|  | | | | | Variance | | S.D |  |  |  |  | Variance | | S.D |
|  |  | Participant (Intercept) | | | 0.18 | | 0.42 |  | Participant (Intercept) | | | 0.16 | | 0.4 |
|  |  |  |  |  |  |  |  |  |  |  |  |  |  |  |
|  |  | Model fit | | | | | |  | Model fit | | | | | |
|  |  | R^2^ | | | Marginal | | Conditional |  | R^2^ | | | Marginal | | Conditional |
|  | | | | | 0.66 | | 0.77 |  |  | | | 0.55 | | 0.69 |
|  | | | | | | | |  |  | | | | | |
| Model equation: reconstruction accuracy ~ group * speech condition + age + hearing + subjective alertness + (1\|participant) | | | | | | | | | | | | | | |
| R package/functions used: Model: lme4/lme; Confidence intervals: stats/confint; p-values: lmerTestllme; R2: MuMIn/r.squaredGLMM | | | | | | | | | | | | | | |

| **Supplementary Table 10: Linear Mixed Effects Modelling of the Impact of Group, Frequency and Speech Condition on Decoding Reconstruction Accuracy** | | | | | | | | | | | | | | | |
| --- | --- | --- | --- | --- | --- | --- | --- | --- | --- | --- | --- | --- | --- | --- | --- |
|  |  | **Model 1: Stimulus Matched: Clear vs. Unintelligible** | | | | | |  | **Model 2: Behaviour Matched: Clear/Low Intelligible vs. Unintelligible** | | | | | | |
|  |  | **Fixed Effects** | | | | | |  | **Fixed Effects** | | | | | | |
|  |  | **Est/Beta** | **SE** | **95% CI** | | **t** | **p** |  | **Est/Beta** | **SE** | **95% CI** | | | **t** | **p** |
| Intercept |  | 0.170 | 0.045 | 0.086 | 0.258 | 3.762 | 0.001 |  | 0.194 | 0.045 | 0.111 | 0.280 | | 4.312 | <0.001 |
| Control vs. Temporal |  | -0.053 | 0.021 | -0.092 | -0.015 | -2.600 | 0.010 |  | -0.034 | 0.021 | -0.074 | 0.005 | | -1.617 | 0.108 |
| Control vs. Frontal |  | -0.029 | 0.021 | -0.069 | 0.011 | -1.364 | 0.175 |  | -0.012 | 0.022 | -0.053 | 0.028 | | -0.554 | 0.580 |
| Frequency (Delta vs. Theta)^1^ |  | -0.078 | 0.018 | -0.115 | -0.047 | -4.366 | <0.001 |  | -0.061 | 0.019 | -0.096 | -0.025 | | -3.269 | 0.001 |
| Speech Condition (Intelligible vs. Unintelligible) |  | -0.081 | 0.018 | -0.112 | -0.044 | -4.550 | <0.001 |  | -0.066 | 0.019 | -0.101 | -0.031 | | -3.573 | 0.001 |
| Group (Control vs. Temporal) x Frequency |  | 0.035 | 0.025 | 0.000 | 0.002 | 1.396 | 0.165 |  | 0.024 | 0.026 | -0.026 | 0.073 | | 0.898 | 0.371 |
| Group (Control vs. Frontal) x Frequency |  | 0.019 | 0.026 | -0.001 | 0.001 | 0.726 | 0.469 |  | 0.007 | 0.027 | -0.044 | 0.058 | | 0.261 | 0.795 |
| Group (Control vs. Temporal) x Speech Condition |  | 0.015 | 0.025 | -0.001 | 0.000 | 0.613 | 0.541 |  | -0.005 | 0.026 | -0.055 | 0.045 | | -0.195 | 0.846 |
| Group (Control vs. Frontal) x Speech Condition |  | 0.003 | 0.026 | -0.033 | 0.063 | 0.099 | 0.921 |  | -0.018 | 0.027 | -0.069 | 0.033 | | -0.676 | 0.500 |
| Frequency x Speech Condition |  | 0.078 | 0.025 | -0.046 | 0.051 | 3.100 | 0.002 |  | 0.066 | 0.026 | 0.017 | 0.116 | | 2.538 | 0.012 |
| Group (Control vs. Temporal) x Frequency x Speech Condition |  | -0.017 | 0.036 | -0.013 | 0.083 | -0.476 | 0.635 |  | -0.005 | 0.037 | -0.076 | 0.065 | | -0.143 | 0.887 |
| Group (Control vs. Frontal) x Frequency x Speech Condition |  | -0.001 | 0.036 | -0.030 | 0.067 | -0.019 | 0.985 |  | 0.011 | 0.038 | -0.061 | 0.083 | | 0.291 | 0.771 |
| Age |  | 0.001 | 0.001 | 0.030 | 0.126 | 1.027 | 0.311 |  | 0.000 | 0.001 | -0.001 | 0.001 | | 0.230 | 0.820 |
| Hearing |  | 0.000 | 0.001 | -0.085 | 0.051 | 0.017 | 0.986 |  | 0.000 | 0.001 | -0.001 | 0.001 | | 0.324 | 0.748 |
| Subjective Alertness |  | -0.001 | 0.000 | -0.070 | 0.068 | -1.324 | 0.194 |  | -0.001 | 0.000 | -0.002 | 0.000 | | -1.916 | 0.063 |
|  | | | | | | | |  |  |  |  | |  |  |  |
|  |  | Random Effects | | | | | |  | Random Effects | | | | | | |
|  | | | | | Variance | | S.D. |  |  |  |  | | Variance | | S.D. |
|  |  | Participant (Intercept) | | | 0.0007 | | 0.03 |  | Participant (Intercept) | | | | 0.0007 | | 0.02 |
|  |  | Experiment Version (Intercept) | | | 0.0002 | | 0.01 |  | Experiment Version (Intercept) | | | | 0.00005 | | 0.007 |
|  |  |  |  |  |  |  |  |  |  |  |  | |  |  |  |
|  |  | Model fit | | | | | |  | Model fit | | | | | | |
|  |  | R^2^ | | | Marginal | | Conditional |  | R^2^ | | | | Marginal | | Conditional |
|  | | | | | 0.26 | | 0.47 |  |  | | | | 0.22 | | 0.42 |
|  | | | | | | | |  |  | | | | | | |
| Model equation: reconstruction accuracy ~ group * frequency * speech condition + age + hearing + subjective alertness + (1\|participant) + (1\|experiment version) | | | | | | | | | | | | | | | |
| R package/functions used: Model: lme4/lme; Confidence intervals: stats/confint; p-values: lmerTestllme; R2: MuMIn/r.squaredGLMM | | | | | | | | | | | | | | | |
| 1. Main effect of frequency is likely to reflect better signal-to-noise and, hence, decoding accuracy for delta than for theta band. | | | | | | | | | | | | | | | |

8.0: Effect sizes measured by Cohen’s *d*

Effect sizes comparing two speech conditions (within-subject) or groups (between-subject) on an outcome measure were calculated using Cohen’s *d*, defined as the mean difference between conditions (collapsing groups) or groups divided by the pooled standard deviation (SD)^34^:

$$\mathrm{Cohen}^{'}s d=\frac{\bar{X_{1}}-\bar{X_{2}}}{SD}$$

$$\mathrm{SD}=\sqrt{\frac{\sum_{g} (n_{g}-1){SD}_{g}^{2}}{\sum_{g} (n_{g}-1)}}$$

where $\bar{X_{1}}$ and $\bar{X_{2}}$ refer to the mean values of the outcome measure in the respective conditions (averaged across all groups) or groups; *n_g_* refers to the number of sample size in the *g*th group; *SD_g_* refers to the standard deviation of *within-subject differences* between the two conditions (for the within-subject Cohen’s *d*) or the standard deviation of the outcome measure (for the between-subject Cohen’s *d*) in the *g*th group. Effect size was considered as small (0.2<Cohen’s *d*<0.5), medium (0.5<Cohen’s *d*<0.8), or large (Cohen’s *d*>0.8)^34^.

9.0: Fisher Z-tests measuring group differences in neural-behavioural relationships

To compare the neural-behavioural relationships (partial correlations between the EEG reconstruction accuracy and d-prime/neuropsychological measures) between groups, the Pearson correlation coefficients (*r*) were first Fisher-transformed into Z-scores:

$$Z=\frac{1}{2}ln(\frac{1+r}{1-r})$$

A test Z-statistic was then measured using the group difference in the Z-score (Z_2_ – Z_1_) divided by the pooled standard error (SE) of the difference35:

$$\mathrm{SE}=\sqrt{\frac{1}{n1-k1-3}+\frac{1}{n2-k2-3}}$$

$$Z_{Statistic}=\frac{Z_{2}-Z_{1}}{SE}$$

where n1 and n2 refer to the respective sample sizes in the two groups; k1 and k2 refer to the respective numbers of the partialled variables in the two groups, where k = 3 (age, hearing and alertness) for the controls and k = 4 (age, hearing, alertness and lesion volume) for the aphasia groups. Statistical significance was quantified using the corresponding p-value (two-tailed) for the test Z-statistic based on the standard normal distribution (with the mean at 0 and standard deviation of 1).

10.0 Filter Response Functions

A second-order zero-phase Butterworth filter was applied to both the broadband speech envelopes and EEG signals at delta (0.5-2 Hz) and theta (2-9 Hz) ranges. Supplementary Figure 1 shows the filter response functions where all cutoff frequencies were at the -3dB roll-off (half power) point.

**Supplementary Figure 1: Filter Response Functions**

**
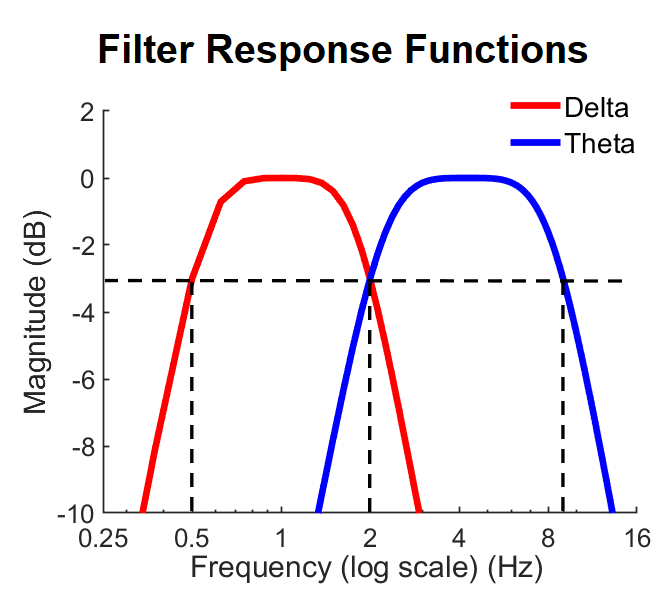
**

Filter response functions for delta (red) and theta (blue) show that all cutoff frequencies (0.5, 2 and 9 Hz, marked by the vertical dashed lines) were at the -3dB roll-off point (i.e., half power of the maximum, marked by the horizontal dashed line).

11.0: Broadband (0.5-9Hz) Envelope Tracking Results

The analyses reported in the main text were replicated using reconstruction accuracies derived from analysis of the broadband (0.5-9Hz) envelope, subsuming our delta and theta ranges.

**Supplementary Figure 2: Normalised Broadband Decoding (left) and Encoding (right) Reconstruction Accuracy**


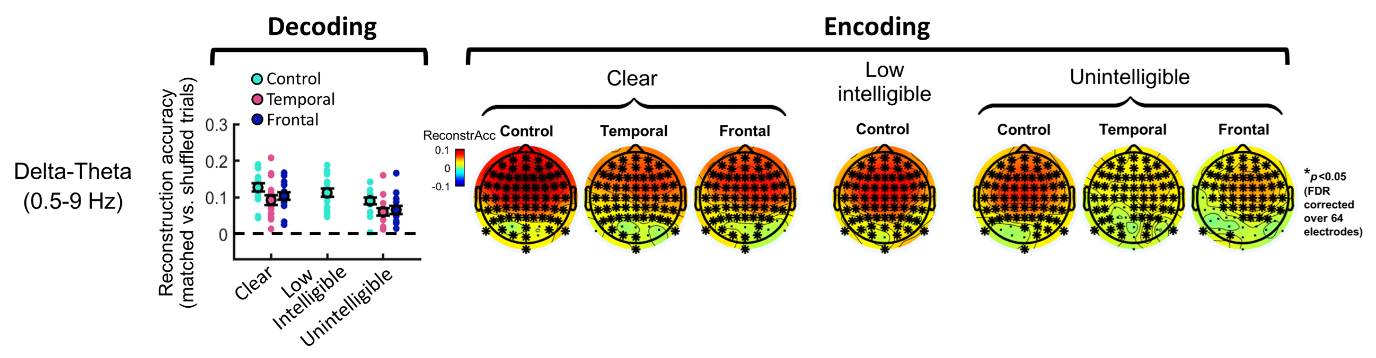


Individual participants’ data are shown for decoding with group mean values in black circles (error bars: ±1 SEM). Dashed lines indicate chance-level accuracy estimated from shuffled trials. Sample sizes: N=15 (temporal), N=14 (frontal) and N=15 (controls). Decoding accuracy was significantly above chance for all groups and speech conditions (one-sample t-tests, t>6.14, p<10^-4^, *p* values uncorrected). Asterisks in the topographs indicate electrodes where encoding reconstruction accuracy for matched trials was significantly greater than for shuffled trials (above chance) (one-sample t-tests, t>2.30, *p*<0.05, FDR-corrected over 64 electrodes).

**Supplementary Figure 3: Statistical Analysis of Stimulus-Matched Broadband Envelope Tracking**


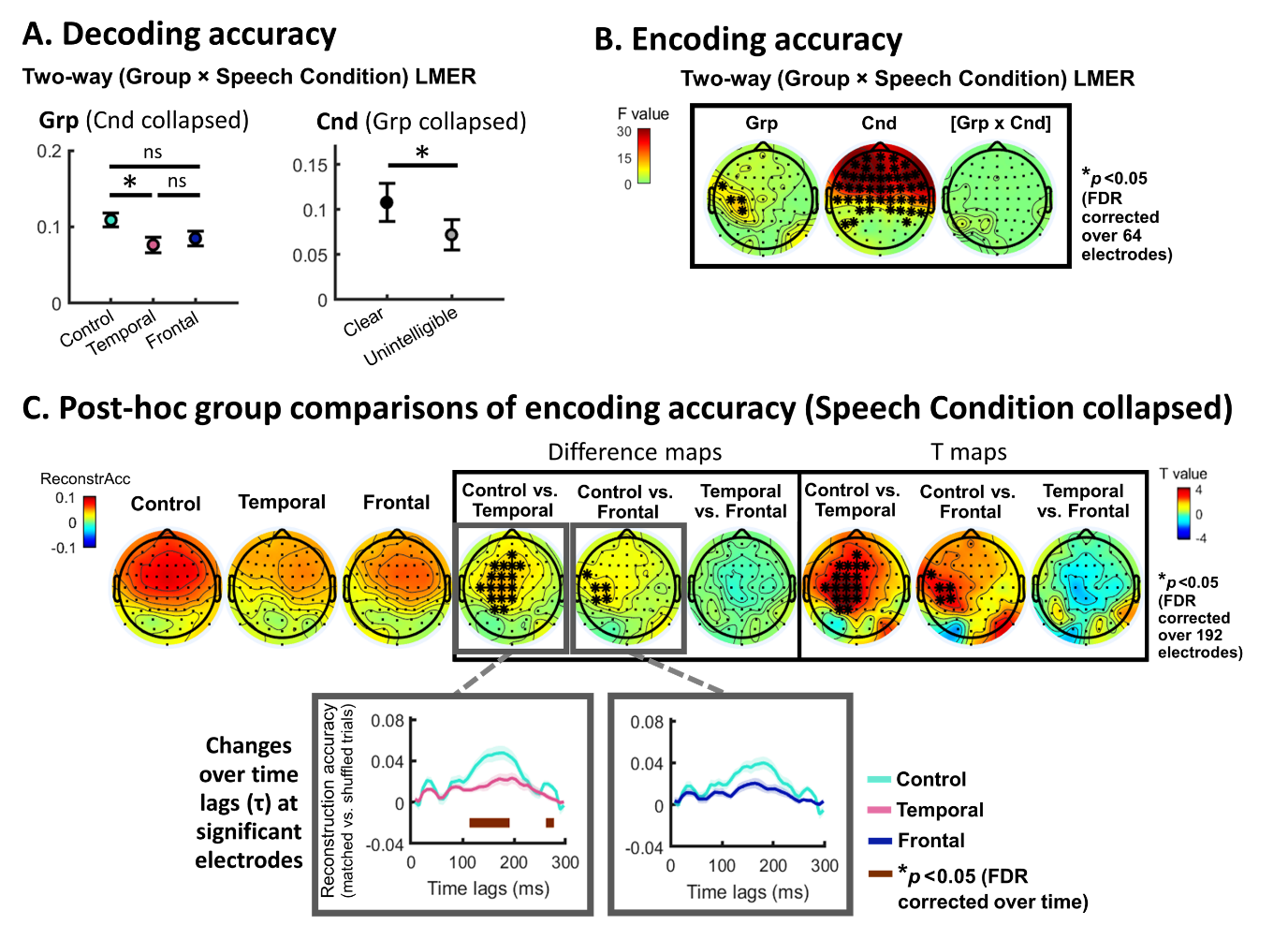


Statistical analysis of decoding and encoding accuracy for speech envelopes in stimulus-matched comparison (clear and unintelligible speech). Sample sizes: N=15 (temporal), N=14 (frontal) and N=15 (controls). **(A) Decoding LMER results.** Two-way (group (Grp) by speech condition (Cnd)) LMER showed: (1) a main effect of group (control>temporal, left panel, t=-2.42, p=0.019) and (2) a main effect of speech condition with greater reconstruction accuracy of clear than unintelligible speech (right panel, t=-3.52, p=0.001). Asterisks indicate significance (p<0.05). ns = non-significance. Error bars indicate ±1 SEM. **(B) Encoding LMER results** demonstrating group and condition effects (intelligible > unintelligible)**.** Asterisks indicate significant electrodes (t>4.6, p<0.05, FDR-corrected for the 64 electrodes). **(C) Encoding model post-hoc comparisons.** Asterisks indicate significant electrodes (independent sample t-tests, t>3.0, *p*<0.05, FDR-corrected for 192 comparisons, i.e., 3 by 64 electrodes). Significantly reduced encoding accuracy in comparison to controls was observed in left central and left hemisphere electrodes in temporal group and in left temporo-parietal electrodes in the frontal group. No differences were observed between the aphasia groups. Bottom panel time-lagged analyses for electrodes with significant group differences. Shaded areas in the time-lagged figures indicate ±1 SEM. Brown lines indicate time-periods during which reconstruction accuracy significantly differed between groups (independent sample t-tests, t>2.6, *p*<0.05, FDR-corrected over time).

**Supplementary Figure 4: Statistical Analysis of Behaviour-Matched Broadband Envelope Tracking**


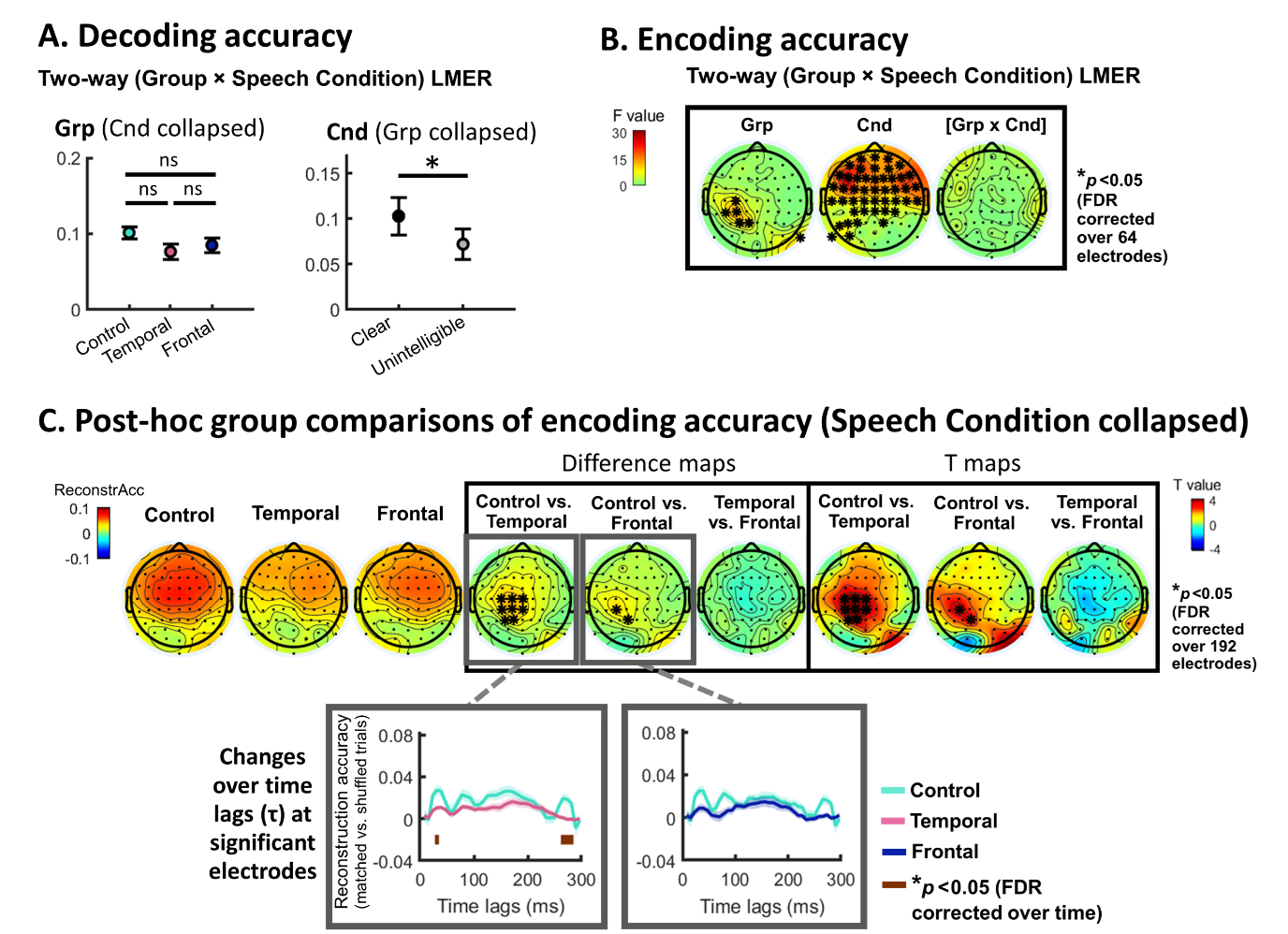


Statistical analyses of decoding and encoding accuracy for behaviour-matched comparison (clear speech in aphasia matched with low-intelligible speech in controls, and unintelligible speech). Sample sizes: N=15 (temporal), N=14 (frontal) and N=15 (controls). **(A) Decoding LMER results** in which borderline condition differences remain (t=-1.97, p=0.056) but group differences observed in the stimulus-matched comparison are no longer significant**. (B) Encoding LMER results** in which group differences and condition differences remain**.** Asterisks indicate significant electrodes (t>4.6, p<0.05, FDR-corrected for the 64 electrodes). **(C) Encoding model post-hoc comparisons.** Asterisks indicate significant electrodes (t>3.3, *p*<0.05, FDR-corrected for 192 comparisons, i.e., 3 by 64 electrodes). Group differences remain in a restricted electrode set in comparison to the stimulus-matched comparison. Bottom panel time-lagged analyses for electrodes with significant group differences. Shaded areas in the time-lagged figures indicate ±1 SEM. Brown lines indicate time-periods during which reconstruction accuracy significantly differed between groups (independent sample t-tests, t>3.1, *p*<0.05, FDR-corrected over time).

**Supplementary Figure 5: Relationship between Broadband Neural Envelope Tracking and d-prime Comprehension Measures from the Intelligible Speech Conditions.**


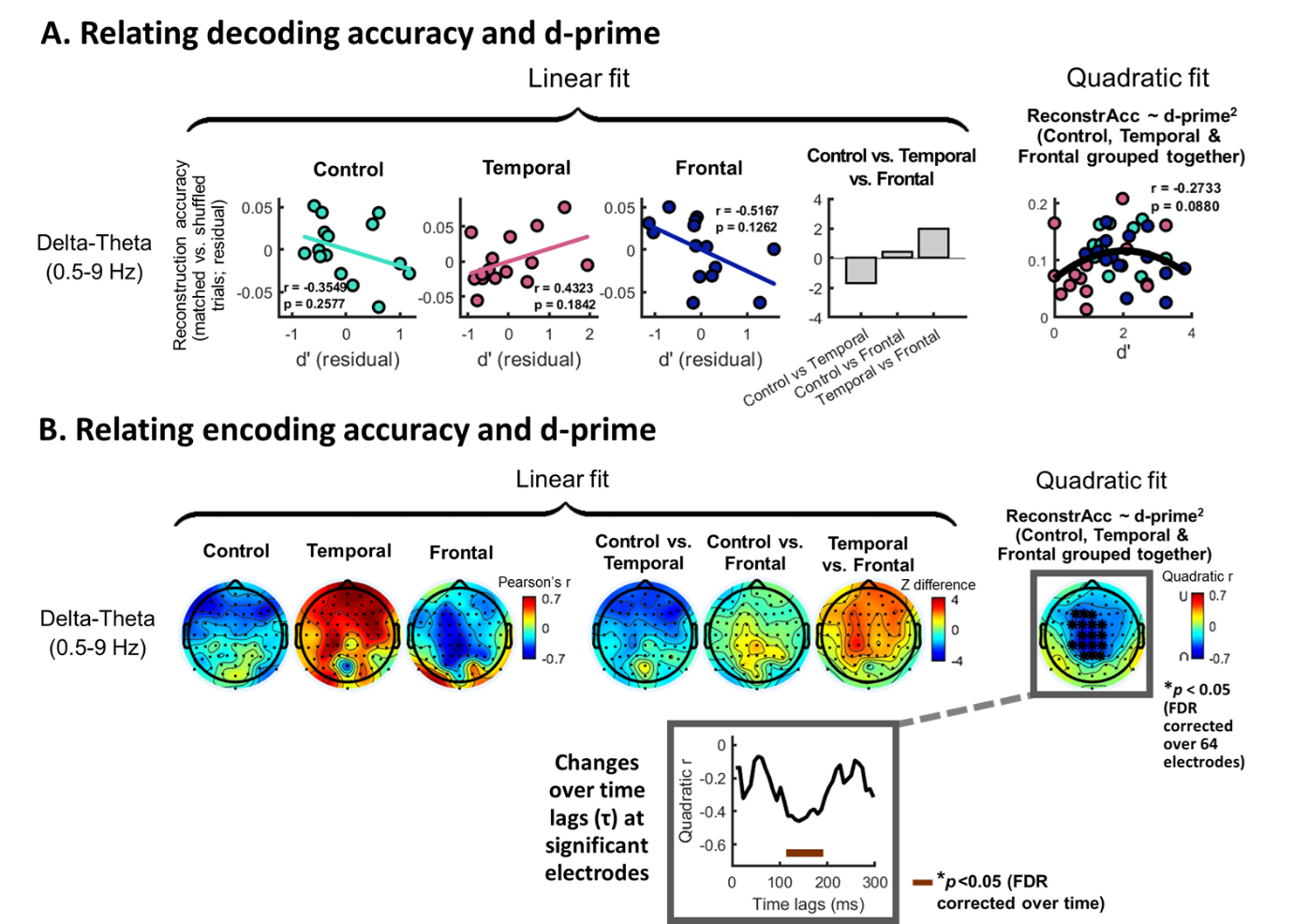


Relationship between broadband neural envelope tracking and d-prime from intelligible speech conditions. Sample sizes: N=15 (temporal), N=14 (frontal) and N=15 (controls). **(A) Decoding accuracy vs. d-prime (d’).** Each dot represents a participant’s data for the residuals of decoding accuracy vs. d-prime (d-prime in the control (green), temporal (pink) and frontal (blue) groups, respectively, after partialling out age, hearing, alertness and lesion volume (aphasia groups only)). Bar graphs (second right panel) show comparison of correlation coefficients between groups. Correlations and group differences are non-significant. **(B) Encoding accuracy vs. d-prime.** For all significant results *p*<0.05, p values were FDR-corrected for 64 electrodes (quadratic r<-0.39 for all significant electrodes) or over time. Significant effects remain in quadratic fit between 100 and 200ms post envelope onset (r<-0.38).

**Supplementary Figure 6: Relationship between Broadband Neural Envelope Tracking from the Intelligible Speech Condition and Neuropsychology Comprehension Summary Score.**


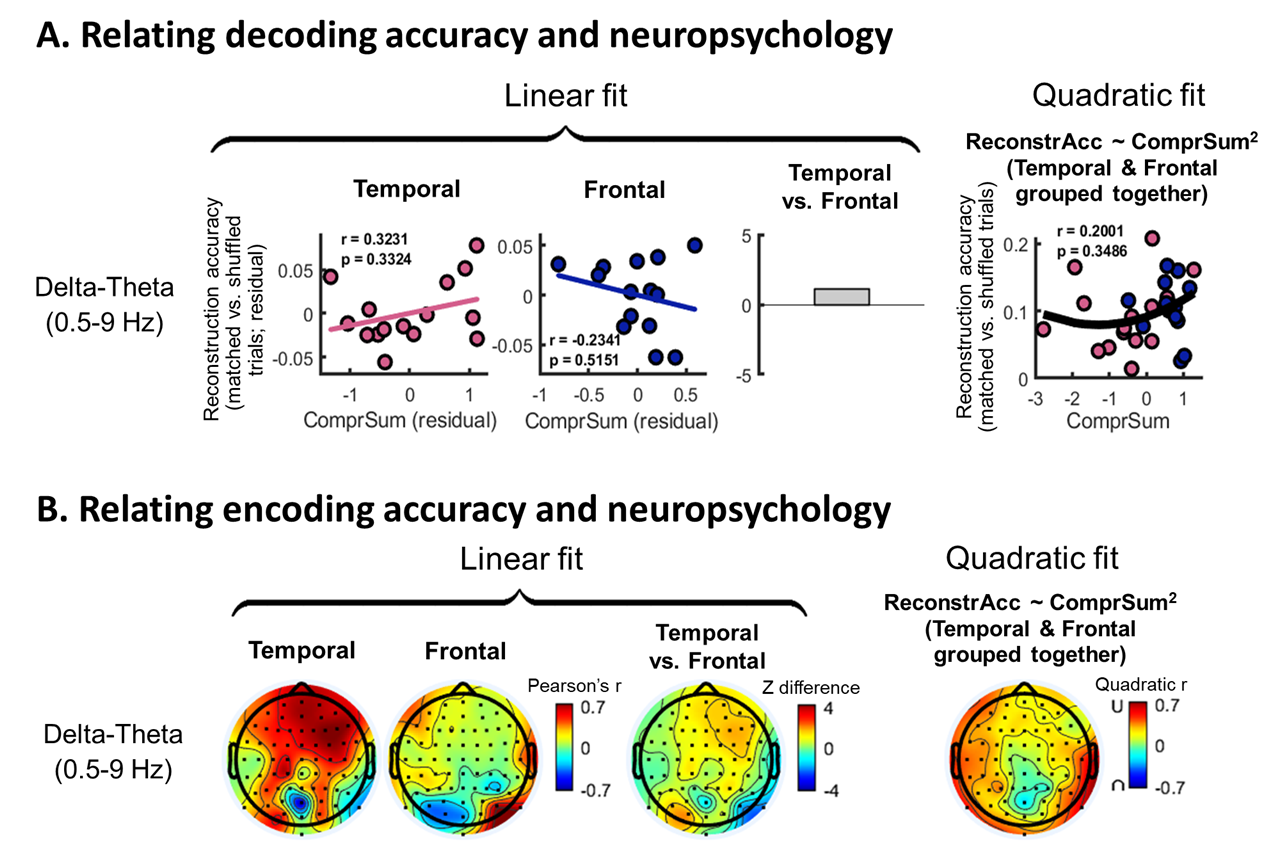


Relationship between broadband neural envelope tracking and neuropsychology measures of comprehension accuracy for aphasia groups (intelligible speech only). Sample sizes: N=15 (temporal) and N=14 (frontal). **(A) Decoding accuracy correlations with summary neuropsychological comprehension scores (ComprSum).** Each dot represents a participant’s data for the residuals of decoding accuracy vs. ComprSum (temporal (pink) and frontal (blue) groups, respectively). Linear partial correlations and quadratic relationship were fit after partialling out age, hearing, alertness and lesion volume. No significant relationship with comprehension or group difference is observed. **(B) Encoding accuracy vs. neuropsychology comprehension summary score (ComprSum).** The significance threshold was based on *p*<0.05 FDR-corrected for 64 electrodes. No significant relationships with comprehension are observed.

12.0: Neural-behavioural relationships for the clear and low-intelligible conditions in the control group

The neural-behavioural relationships (correlations of envelope tracking with d-prime) were shown to be highly similar between the two intelligible (clear and low-intelligible) speech conditions in the control group (see Supplementary Figure 7). Therefore, we collapsed the two conditions for this analysis for the controls.

**Supplementary Figure 7: Relationship between Broadband Neural Envelope Tracking and the d-prime Comprehension Measures from the Two Intelligible Speech Conditions for the Control Group.**

**
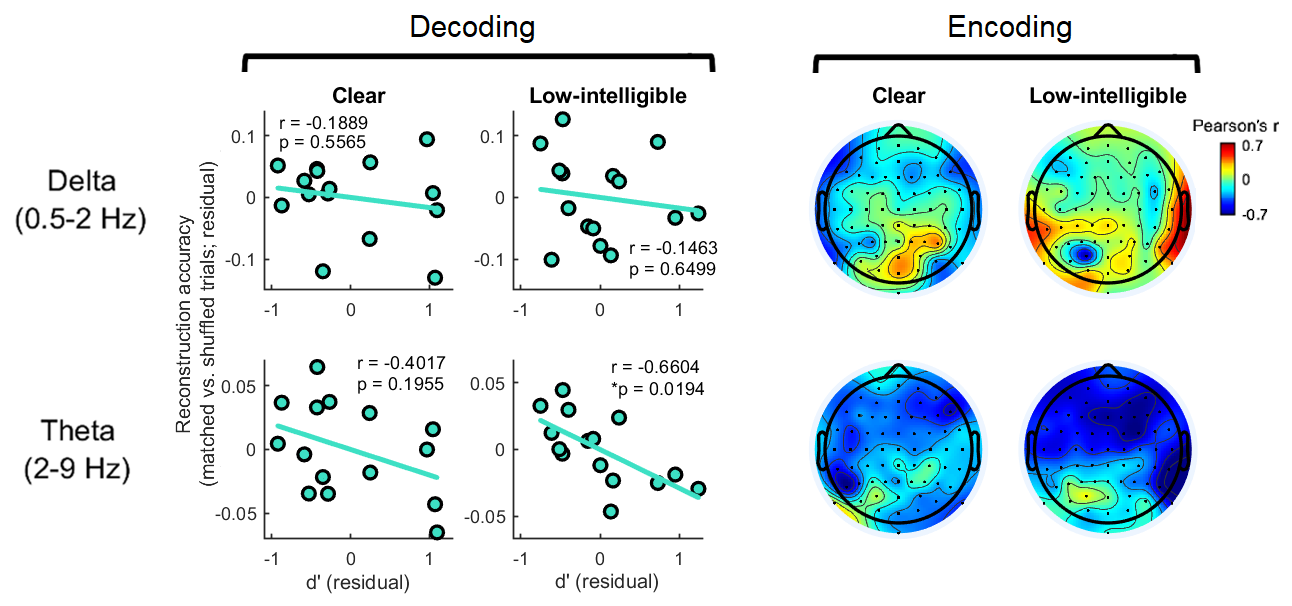
**

Partial correlations of decoding (left) and encoding (right) accuracy with d-prime (d’) from the clear and low-intelligible speech conditions for the controls after partialling out age, hearing, alertness. Sample size: N=15 (control). Each dot (left, decoding) represents a participant’s data for the residuals of decoding accuracy vs. d-prime. The correlations were highly similar between the two conditions for both decoding and encoding. A consistent pattern of highly negative correlations in the theta band was observed, in line with that in Figure 5 in the main text.

13.0: Non-parametric tracking-comprehension relationships.

Spearman’s correlations between theta envelope tracking and comprehension measures were performed to ensure effects were not driven by outliers. The overall pattern of results was consistent with the parametric correlations reported in the main text.

| **Supplementary Table 11: Spearman’s Correlations – Theta rate envelope tracking vs. comprehension** | | |
| --- | --- | --- |
| **Group** | **D-prime (EEG paradigm)** | **Summary Neuropsychological Comprehension Scores** |
| Control | ρ=0.5036, p=0.0582 |  |
| Temporal | ρ =0.8071, p=0.0004 | ρ=0.8250, p=0.0002 |
| Frontal | ρ =-0.4110, p=0.1458 | ρ =-0.7785, p = 0.0017 |

**Supplementary References**

1. Robson H, Bose A, Meteyard L. DALIP Research Registry. 2015. https://www.hra.nhs.uk/planning-and-improving-research/application-summaries/research-summaries/dalip-research-registry/

2. Seghier ML, Patel E, Prejawa S, et al. The PLORAS database: a data repository for predicting language outcome and recovery after stroke. *Neuroimage*. 2016;124:1208-1212.

3. Goodglass H, Kaplan E, Barresi B. *The Assessment of Aphasia and Related Disorders*. Lippincott Williams & Wilkins; 2001.

4. Nasreddine ZS, Phillips NA, Bédirian V, et al. The Montreal Cognitive Assessment, MoCA: a brief screening tool for mild cognitive impairment. *J Am Geriatr Soc*. 2005;53(4):695-699.

5. van Buuren S, Groothuis-Oudshoorn K. mice: Multivariate Imputation by Chained Equations in R. *J Stat Softw*. 2011;45(3):1-67. doi:10.18637/jss.v045.i03.

6. Robson H, Pilkington E, Evans L, DeLuca V, Keidel JL. Phonological and semantic processing during comprehension in Wernicke’s aphasia: An N400 and Phonological Mapping Negativity Study. *Neuropsychologia*. 2017;100:144-154. doi:http://dx.doi.org/10.1016/j.neuropsychologia.2017.04.012

7. Nasreddine ZS, Phillips NA, Bédirian V, et al. The Montreal Cognitive Assessment, MoCA: a brief screening tool for mild cognitive impairment. *J Am Geriatr Soc*. 2005;53(4):695-699.

8. van Buuren S, Groothuis-Oudshoorn K. mice: Multivariate Imputation by Chained Equations in R. *J Stat Softw*. 2011;45(3):1-67. doi:10.18637/jss.v045.i03.

9. Robson H, Thomasson H, Davis MH. Designing remote synchronous auditory comprehension assessment for severely impaired individuals with aphasia. *Int J Lang Commun Disord*. Published online 2023.

10. Manly T, Davison B, Heutink J, Galloway M, Robertson IH. Not enough time or not enough attention? Speed, error and self-maintained control in the Sustained Attention to Response Test (SART). *Clinical Neuropsychological Assessment: An iIternational Journal for Research & Clinical Practice*. 2000;3:167-177.

11. Anwyl-Irvine AL, Massonnié J, Flitton A, Kirkham N, Evershed JK. Gorilla in our midst: An online behavioral experiment builder. *Behav Res Methods*. 2020;52:388-407.

12. Swinburn K, Porter G, Howard D. Comprehensive aphasia test. Published online 2004.

13. Robson H, Thomasson H, Upton E, Leff AP, Davis MH. The impact of speech rhythm and rate on comprehension in aphasia. *Cortex*. 2024;180:126-146.

14. Kertesz A. *Western Aphasia Battery--Revised*. Psychological Corporation; 2007.

15. Brookshire RH, Nicholas LE. *Discourse Comprehension Test*. Communication Skill Builders; 1993.

16. Mackenzie C. The relevance of education and age in the assessment of discourse comprehension. *Clin Linguist Phon*. 2000;14(2):151-161.

17. Kay J, Lesser R, Coltheart M. *PALPA: Psycholinguistic Assessments of Language Processing in Aphasia*. Psychology Press; 2009.

18. Davis MH, Evans S, McCarthy K, Evans L, Giannakopoulou A, Taylor J. Lexical learning shapes the development of spech perception until late adolescence. *PsyArXiv Preprints*. Published online 2019.

19. Bozeat S, Lambon Ralph MA, Patterson K, Garrard P, Hodges JR. Non-verbal semantic impairment in semantic dementia. *Neuropsychologia*. 2000;38(9):1207-1215. doi:http://dx.doi.org/10.1016/S0028-3932(00)00034-8

20. Wechsler D. WAIS-R : Wechsler adult intelligence scale-revised. *Psychological Corporation*. Preprint posted online 1981.

21. Burgess PW, Shallice T. *The Hayling and Brixton Tests*. Thames Valley Test Company; 1997.

22. Manly T, Davison B, Heutink J, Galloway M, Robertson IH. Not enough time or not enough attention? Speed, error and self-maintained control in the Sustained Attention to Response Test (SART). *Clinical Neuropsychological Assessment: An International Journal for Research & Clinical Practice*. 2000;3:167-177.

23. Hunt R, Brychta A. *Biff, Chip and Kipper Series*. Oxford University Press

24. Hunt R, Hunt D, Brychta A. *The Big Breakfast*. Oxford University Press; 2014. https://books.google.co.uk/books?id=TcjawQEACAAJ

25. Oxford University Press. *The Lightning Key*. Oxford University Press; 2018.

26. Oxford University Press, Brychta A. *The Time Capsule*. Oxford University Press; 2015. https://books.google.co.uk/books?id=9FcAogEACAAJ

27. Verschueren E, Vanthornhout J, Francart T. The effect of stimulus intensity on neural envelope tracking. *Hear Res*. 2021;403:108175.

28. Peelle JE, Gross J, Davis MH. Phase-locked responses to speech in human auditory cortex are enhanced during comprehension. *Cerebral Cortex*. 2013;23(6):1378-1387.

29. Greenwood DD. A cochlear frequency‐position function for several species—29 years later. *J Acoust Soc Am*. 1990;87(6):2592-2605. doi:10.1121/1.399052

30. Tadel F, Baillet S, Mosher JC, Pantazis D, Leahy RM. Brainstorm: A User-Friendly Application for MEG/EEG Analysis. *Comput Intell Neurosci*. 2011;2011(1):879716. doi:https://doi.org/10.1155/2011/879716

31. Crosse MJ, Di Liberto GM, Bednar A, Lalor EC. The multivariate temporal response function (mTRF) toolbox: a MATLAB toolbox for relating neural signals to continuous stimuli. *Front Hum Neurosci*. 2016;10:604.

32. Haro S, Rao HM, Quatieri TF, Smalt CJ. EEG alpha and pupil diameter reflect endogenous auditory attention switching and listening effort. *European Journal of Neuroscience*. 2022;55(5):1262-1277.

33. Wechsler D. WAIS-R : Wechsler adult intelligence scale-revised. *Psychological Corporation*. Preprint posted online 1981.

34. Cohen J. Statistical power analysis for the behavioral sciences. *Routledge*. 2013.

35. Hinkle DE, Wiersma W, Jurs SG. *Applied Statistics for the Behavioral Sciences*. Vol 663. Houghton Mifflin Boston; 2003.
